# The nucleus accumbens as a nexus between values and goals in goal-directed behavior: a review and a new hypothesis

**DOI:** 10.3389/fnbeh.2013.00135

**Published:** 2013-10-23

**Authors:** Francesco Mannella, Kevin Gurney, Gianluca Baldassarre

**Affiliations:** ^1^Laboratory of Computational Embodied Neuroscience, Institute of Cognitive Sciences and Technologies, National Research CouncilRome, Italy; ^2^Department of Psychology, The University of SheffieldSheffield, UK

**Keywords:** goal-directed Behavior, goal selection, value, novelty, amygdala, hippocampus, nucleus accumbens, prefrontal cortex

## Abstract

Goal-directed behavior is a fundamental means by which animals can flexibly solve the challenges posed by variable external and internal conditions. Recently, the processes and brain mechanisms underlying such behavior have been extensively studied from behavioral, neuroscientific and computational perspectives. This research has highlighted the processes underlying goal-directed behavior and associated brain systems including prefrontal cortex, basal ganglia and, in particular therein, the nucleus accumbens (NAcc). This paper focusses on one particular process at the core of goal-directed behavior: how motivational value is assigned to goals on the basis of internal states and environmental stimuli, and how this supports goal selection processes. Various biological and computational accounts have been given of this problem and of related multiple neural and behavior phenomena, but we still lack an integrated hypothesis on the generation and use of value for goal selection. This paper proposes an hypothesis that aims to solve this problem and is based on this key elements: (a) amygdala and hippocampus establish the motivational value of stimuli and goals; (b) prefrontal cortex encodes various types of action outcomes; (c) NAcc integrates different sources of value, representing them in terms of a common currency with the aid of dopamine, and thereby plays a major role in selecting action outcomes within prefrontal cortex. The “goals” pursued by the organism are the outcomes selected by these processes. The hypothesis is developed in the context of a critical review of relevant biological and computational literature which offer it support. The paper shows how the hypothesis has the potential to integrate existing interpretations of motivational value and goal selection.

## 1. Introduction

Instrumental learning—the process of acquiring the capacity to select actions based on the utility of their outcomes—is a fundamental means through which animals adapt to changes in their environment. These changes may be profound, as the ecological niches occupied by animals can vary substantially during the life of a single individual. For example, the superficially straightforward behaviors of foraging, escaping predators, and searching for con-specifics, must be flexible and dynamically adjust to continuously changing environmental conditions. Moreover, action selection processes have to flexibly adjust on the basis of internal states and needs of the animal, as these continuously change in the course of the day. Only when there are strong invariances in the contingencies between action and valuable outcomes, based on reliable environmental and internal processes, behavior can become more regular or *habitual*. When this is not possible, the selection of instrumental actions is based on the current value of action outcomes, or *goals* (Balleine and Dickinson, 1998). Here we use the term *goal* to indicate the *internal representation* of an action *outcome* currently chosen as the target of the animal's behavior because of the *incentive salience*, or *motivational value*, associated with the outcome. Incentive salience has been defined as a motivational attribute that the brain assigns to stimuli if these are related to the possible satisfaction of some of the animal's homeostatic drives (Berridge, 2004; we shall see how our hypothesis expands the concept of motivational value to include value related to the novelty of outcomes). The theory proposed here is hence relevant for decisions that involve the selection of action goals on the basis of their current value for the animal, in particular “ultimate goals” consisting in the achievement or interaction with items having an intrinsic biologically saliency (e.g., food, water, and novel objects). When behavior is sensitive to the value assigned to goals and to the contingencies between goals and actions that can accomplish them, it is referred to as *goal-directed behavior* (Dickinson and Balleine, 1994; Balleine and Dickinson, 1998).

The processes and neural mechanisms through which the brain generates and assigns motivational incentive salience to goals is an important open problem for current neuroscientific research: this paper addresses this issue by first offering a critical review of the relevant literature, and then by proposing a novel hypothesis to solve it. The biological literature indicates that goal-directed behavior rests on the acquisition of two key types of associations: first, the associations between actions and their outcomes which have to be learned so that the animal can choose actions when their outcomes become desirable (Balleine and Dickinson, 1998; Yin and Knowlton, 2006); second, the associations between the outcomes and their current motivational value (Balleine and Dickinson, 1998; Balleine and Killcross, 2006; Yin and Knowlton, 2006; Balleine and Ostlund, 2007). Instrumental behaviors that do not rely on these two classes of associations are deemed *habitual*, being solely based on associations between stimuli and responses (S-R; Balleine and Dickinson, 1998; Yin and Knowlton, 2006).

The neural substrates underlying goal-directed and habitual behavior have been extensively investigated, and several key neural systems have been shown to be involved. These include the basal ganglia, a set of subcortical nuclei which form looped circuits with cortex and thalamus. The main input nucleus to the basal ganglia is the striatum, which may be partitioned, on an anatomical basis, into dorsolateral, dorsomedial and ventral territories. It is the ventral striatum, otherwise known as nucleus accumbens (NAcc), which forms a focus of the paper. The accumbens is further divided into two major sub-components: the “core” and the “shell.” Other key areas for goal-directed behavior include the limbic structures such as the amygdala and hippocampus. The amygdala is a brain system that, along with others (e.g., hypothalamus), helps homeostatic regulation of internal bodily organs (e.g., heart rate and blood pressure) and of the neuromodulators in the brain, and affects the triggering of innate behaviors such as orienting and approach. Hippocampus is a highly-associative multimodal part of the “paleocortex” (phylogenetically older than neocortex) and is strongly connected with all associative cortical areas; it plays an important role in episodic memory, consolidation of long-term memory, and higher-level cognition (e.g., planning).

While the anatomical identity of these key structures has been established, there is, as yet, no complete picture of their operation in behavioral expression, although some broad functional separation can be made. Thus, it appears that habitual behavior, and related learning processes, are rooted in the circuits involving the dorsolateral striatum and motor cortex (Packard and Knowlton, 2002; Yin et al., 2004). In contrast, goal-directed behavior relies on the networks including prefrontal cortex, dorsomedial striatum, and NAcc portions of basal ganglia, and limbic neural structures such as amygdala and hippocampus (Corbit et al., 2001; Yin et al., 2005).

Recently, the theoretical work on these issues has been corroborated by studies based on computational models and formal analyses. In particular, several concepts of the reinforcement learning (Sutton and Barto, 1998) and optimal control theory have been exploited to formally capture various features and differences of goal-directed and habitual instrumental behavior. For example, in a seminal paper, Daw et al. (2005) proposed that habitual behavior and its learning can be captured on the basis of *model-free reinforcement learning*, whereas the functionalities involved in goal-directed behavior can be represented through *model-based reinforcement learning*.

Within this framework, a key role has been ascribed to the NAcc in terms of processing of current values and reward predictions (Humphries and Prescott, 2010; Bornstein and Daw, 2011; Penner and Mizumori, 2011; Pennartz et al., 2011; Khamassi and Humphries, 2012). However, the role played by the NAcc in the interaction between values and outcomes has not been fully clarified. The NAcc, in synergy with amygdala, has been shown to play an important role also in Pavlovian (classical conditioning) processes, responsible for assigning value to previously neutral stimuli (Corbit et al., 2001; Cardinal et al., 2002b; Day et al., 2006; Day and Carelli, 2007; Yin et al., 2008; Lex and Hauber, 2010; Mannella et al., 2010). These processes have also been shown to produce “energizing” effects on instrumental behavior, e.g., causing lever pressing with higher strength and frequency, based on the value assigned to the stimulus (this phenomenon is known as “Pavlovian to Instrumental Transfer”—PIT; Corbit et al., 2001; Hall et al., 2001; Corbit and Balleine, 2011). Notwithstanding this evidence, the key contribution of NAcc to assign value to goals in goal-directed processes has not been fully spelled out. Furthermore, while the contribution of the amygdala-accumbens system is known to be important when appetitive and aversive motivational values are involved, a possible role of the hippocampal projection to accumbens in supplying goal-value has still not been clarified. It is known that the hippocampus plays a key role in goal processing (Pennartz et al., 2011) and also in the detection of the salience of stimuli based on their *novelty* (Lisman and Grace, 2005).

Our account seeks to unify these observations under the idea that NAcc serves to integrate different types of value sources used to select goals. This perspective will specify and articulate in a new way the classic idea that the NAcc acts as an interface between “the limbic system and the motor system” of brain (Mogenson et al., 1980). In particular, in this paper we propose an integrated system-level hypothesis to explain how various types of motivational values are transferred to goals via the NAcc, and how goals, in turn, control instrumental behavior. The hypothesis also explains the role played by the projections of amygdala and hippocampus to NAcc in defining different types of value, in particular values related to appetitive, aversive, and novel stimuli (although we will deal with aversive stimuli only marginally).

The basic idea is that Pavlovian processes in amygdala, and novelty-detection processes in hippocampus, are capable of assigning motivational value to biologically relevant stimuli and events. The NAcc collects information on motivational value from disparate sources and encodes it in an integrated way in the “common currency” of its activity. This information is then used, via ventral basal ganglia connectivity to prefrontal cortex, to select among possible future goals encoded there. Further, we propose two mechanisms for this. The first involves accumbens core which contributes to goal selection with the same mechanisms used by other striatal territories to make selections: competition between alternative options and disinhibition of thalamic targets (in this case representing goals), by basal ganglia output nuclei. In contrast, a second mechanism involving accumbens shell exploits its strong connections with the dopaminergic system to make goal selection “promiscuous.” That is, increased dopamine (DA) in accumbens shell makes selection possible with smaller input salience. This “softer” selection scheme may have two possible functions. First, it could foster goal exploration during learning phases and the parallel selection of multiple related goals and sub-goals during exploitation. Second, it could support a more effective summation of the value of outcomes, and hence a better comparison of them, when multiple sources of such value are available at the same time.

Cortex also encodes actions required to bring about the selected goals, and the associations between outcomes and actions that allow the selected goals to activate the representations of specific actions. These are *internal models*, specifically *inverse models* (action-to-outcome), usable for action deployment (Gurney et al., 2012). The action representations excited by goals through inverse models are subject to selection via basal ganglia, so allowing their behavioral expression and hence the achievement of the goals that activated them.

The rest of the paper expands the hypothesis as follows. Section 2 presents a focussed review of some main bio-behavioral neuroscientific proposals and computational models aiming to explain the learning and expression of goal-directed behavior. Based on this, section 3 first presents an evolutionary interpretation of the functions and neural structures underlying goal-directed behavior: this is a framework within which we develop the core hypothesis proposed here. The section continues by explaining the neural basis of the hypothesis. In particular it describes the three main components of the hypothesis: (1) the amygdala and hippocampus as the sources of motivational value, (2) the ventral basal ganglia (including NAcc) as the sub-system integrating motivational-value information and selecting goals on this basis, (3) the prefrontal cortex as the main component representing and predicting outcomes, and triggering the execution of actions that lead to these outcomes, based on action-outcome contingency representations. Finally, section 4 draws conclusions, in particular highlighting how the proposed hypothesis reconciles most functions attributed in the literature to NAcc. The acronyms used in the paper are listed in Table A1.

## 2. Goal-directed behavior: current biological and computational frameworks

### 2.1. Goal-directed behavior: key functional processes

The definition of goal-directed behavior is based on two behavioral effects and the experimental paradigms to investigate them, namely contingency degradation and instrumental devaluation. In a typical *contingency degradation* experiment (e.g., Balleine and Dickinson, 1998) an animal first learns to produce an instrumental action (e.g., a lever press) to obtain a reward (e.g., a food pellet). After this training the same reward is presented to the animal independently of the production of the action, so degrading the correlation (“contingency”) between the performance of the action and the experience of its outcome. After the contingency degradation, the animal exhibits a lower probability of performing the instrumental action. These results indicate that, throughout the instrumental training, the animal learns and continuously updates the association between action and outcome. This association is then used to select the current action based on the chosen outcome. In a typical *instrumental devaluation* experiment (e.g., Balleine and Dickinson, 1998) an animal first learns to obtain two rewards (e.g., a food pellet and a sucrose solution) via two instrumental actions (e.g., pressing a lever and pulling a chain). Then one of the rewards is devalued, for example by letting the animal freely access it until satiation. In a subsequent test where both manipulanda (lever and chain) are presented together “in extinction”—that is *without* rewards—the animal tends to perform with lower probability the instrumental action corresponding to the devalued outcome.

**Figure 1:**
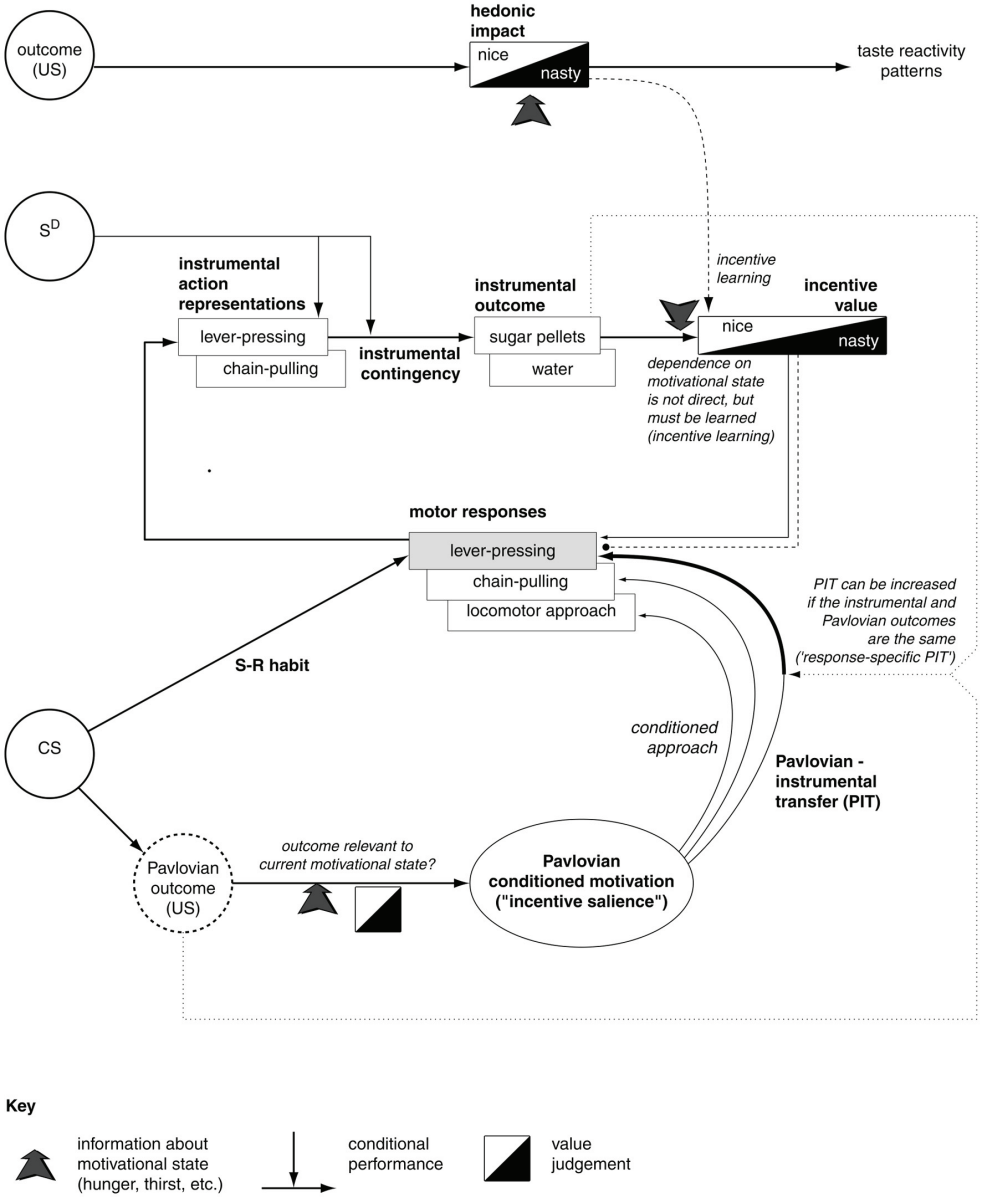
**The major associations and processes behind instrumental habitual behavior, goal-directed behavior, Pavlovian processes, and their relations (here we describe only those relevant for this work)**. At the top, the diagram shows the systems processing the hedonic impact and the incentive value of stimuli, the latter important for the assignment of value to appetitive/aversive stimuli involved in goal-directed processes. The middle of the diagram shows the loop of processes involving goal-directed behavior; here the action representations are associated with outcome representations (instrumental contingency) and then these outcomes are attributed incentive value. In this way, outcomes can trigger the execution of motor responses that lead to them. The bottom of the diagram refers to Pavlovian processes, with the core association between conditioned stimuli (CS) to unconditioned stimuli (US). These have a certain value depending on the animal's internal states. Pavlovian processes can directly trigger unlearned behaviors (e.g., as in conditioned approach experiments) or influence the performance of instrumental behaviors (Pavlovian-Instrumental Transfer—PIT). The diagram also represents the formation of habits (S-R behaviors) as direct associations between stimuli (CS) and motor responses. Reprinted from Cardinal et al. (2002a), Copyright 2002, with permission from Elsevier.

Together, the experiments of contingency degradation and devaluation capture the core functional processes behind goal-directed behavior. Figure 1 summarizes the main ideas in the literature related to the interpretation of the mechanisms underlying these processes and their relation to S-R/habitual behaviors (e.g., see Dickinson and Balleine, 1994; Balleine and Dickinson, 1998; Cardinal et al., 2002a). We now illustrate these processes in detail.

The first set of processes involve the attribution of value to stimuli during *consummatory behaviors* (related to “liking” Berridge, 2004). These processes have overt behavioral manifestations and, according to the literature, might be related to mechanisms that are responsible for the attribution of value to outcomes in goal directed behavior. The second class of phenomena involve the complex processes behind *goal-directed behavior* (Dickinson and Balleine, 1994; Balleine and Dickinson, 1998). These processes are related to the representation of the associations between action representations and outcome representations (instrumental contingency) investigated in contingency degradation experiments. These mechanisms are also related to the attribution of incentive value to outcomes and the consequent recall of suitable motor responses—the processes investigated in devaluation experiments. The third class of phenomena is related to *Pavlovian processes*, involving the core associations between conditioned stimuli (CS) and unconditioned stimuli (US; Cardinal et al., 2002a). The US have a value depending on the animal's internal states. Pavlovian processes can directly trigger unlearned behaviors (e.g., as in conditioned approach experiments) or influence the performance of instrumental behaviors (Pavlovian-Instrumental Transfer—PIT; Corbit et al., 2007).

If instrumental actions are repeated a great number of times in constant conditions (“overtraining”) the behavior tends to become insensitive to the value of goals (McDonald and White, 1993; Yin and Knowlton, 2006). In this case, the associations between the perceived stimulus/overall context and the produced responses (S-R) are so strongly encoded that behavior becomes *habitual*, i.e., mainly guided by external stimuli alone.

We will show that, with respect to the views described above, our hypothesis presents three important new ideas. First, it proposes that aside from *appetitive value*, driving both instrumental and Pavlovian processes (and originating mainly from amygdala–Amg), a second important source of value is used to select goals, namely, “intrinsic value” related to the *novelty* of stimuli, and originating from the hippocampus (Hip). Second, it specifies the mechanisms of attribution of value to goals, in particular, spelling out the mechanisms through which value is generated and contributes to goal selection. Third, it highlights the importance of the representation of “inverse models” (where the activation of a goals/outcomes triggers the recall of actions) instead of the more usually emphasized “forward” or “prediction models” (where the information flow goes from “instrumental action representations” to “instrumental outcome,” see Figure 1); see Gurney et al. (2012), on the distinction between the two types of models.

**Figure 2:**
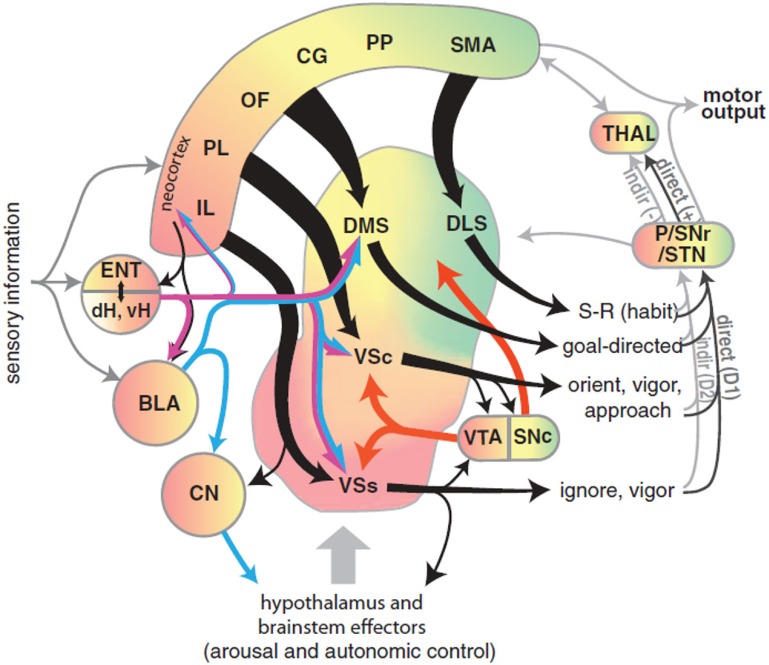
**Diagram of the rat brain illustrating a proposal on the role of DLS to select habits, of DMS to drive goal-directed behavior, and of NAcc to assign vigor to the performance of selected behaviors and to trigger ancillary behaviors such as orienting and approaching**. Reprinted with permission (Gruber and McDonald, 2012).

Recently, Gruber and McDonald (2012) have proposed an integrated theory on the brain system underlying goal-directed behavior dealing with some of the ideas described above. This theory has some similarities with, but also important differences from, our proposal (Figure 2). As we do here, Gruber and McDonald (2012) relate the dorsolateral striatum (DLS, in rats; “putamen” in primates) to habits and sensorimotor behavior. However, in contrast to our theory, the main processes involved in goal selection are ascribed to the dorsomedial striatum (DMS, in rats; “caudatum” in primates) and not to the NAcc. The latter is, instead, supposed to implement supportive functions such as the regulation of “energization” or “vigor” of the performed behaviors, and the triggering of behaviors which are ancillary to the main instrumental behavior (for example orienting and approaching). This proposal is part of a literature that tends to closely associate goal-directed behavior to DMS and to ascribe a motivational/supportive role to NAcc (Yin and Knowlton, 2006; Balleine et al., 2007, 2008). Although very relevant, these proposals do not fully explain, as our model does, how information on the ultimate cause of goal selection, namely value, is *transmitted* to goals. Equally important, the proposal does not fully explain where value *originates*. For example, the proposal does not explain why, in instrumental devaluation experiments, NAcc is necessary to allow a rat to decide which lever to press, given two levers instrumentally associated with two different foods, on the basis of the value currently assigned to such foods. Moreover, the proposal does not fully articulate how such value, both appetitive and related to novelty, is generated. Thus it would have difficulty in explaining why the basolateral amygdala (BLA) is necessary for the production of the devaluation effects. Our proposal explains these results and also reconciles them with the functions that Gruber and McDonald (2012) and similar proposals ascribe to NAcc. In particular, our proposal claims that: (a) In early stages of evolution, NAcc learned to play an important role in Pavlovian processes triggering a number of innate behaviors such as those related to orienting, approaching, and avoidance (see section 3.1 for details). (b) The NAcc encodes outcome value originating from different sources with a unique “currency” (namely, the activation of the representations of the possible outcomes themselves). This value representation is intimately related to DA, as NAcc is one of the main regulators of, and targets for, DA production. Such DA production is one of the main physiological correlates of vigor transferred by NAcc to lower-level striatal regions implementing actions (see section 3.5). (c) With the later evolutionary expansion of prefrontal cortex (PFC) in mammals and, hence the potentiation of their capacity to form, represent, and manage goals, the NAcc has acquired a prominent role in the assignment of value to such goals; this is the main function of NAcc expanded in this paper (see sections 3.1 and 3.5). (d) The goals selected on the basis of these mechanisms then contribute to select the actions to be performed via the top-down control exerted by the NAcc-PFC system on the lower-level associative and sensorimotor striato-cortical systems via cortical and sub-cortical inverse models (see section 3.6). The DMS plays a key role in the latter process and in the specification of sub-goals, hence its importance for learning and expressing goal-directed behavior.

### 2.2. Goal-directed behavior: neural correlates

Instrumental goal-directed behavior is supported by both the activity of cortical regions and the activity of various subcortical neural components. Balleine et al., (2009) summarize the most important neural regions needed for the acquisition and/or expression of instrumental devaluation and contingency degradation in rats based on the effect of lesions on the two behavioral effects. Among these, lesions of the prelimbic cortex (PL; Corbit and Balleine, 2003), the DMS (Yin et al., 2005), or the mediodorsal thalamus (Corbit et al., 2003; Ostlund and Balleine, 2008) result in a lack of expression of both effects. In contrast, lesions of the orbitofrontal cortex (OFC; Ostlund and Balleine, 2007a,b) or the entorhinal cortex (EC) result in a lack of expression of contingency degradation alone (but not so in primates where OFC is important for instrumental devaluation, Euston et al., 2012; Roberts, 2006). Finally, lesions of the NAcc, in particular of its core sub-component (NAccCo; Corbit and Balleine, 2003) results in a lack of expression of instrumental devaluation alone. Importantly, the acquisition and expression of instrumental devaluation is also disrupted by a damage at the level of the Amg, in particular the BLA (Balleine et al., 2003; Mannella et al., 2010).

**Figure 3:**
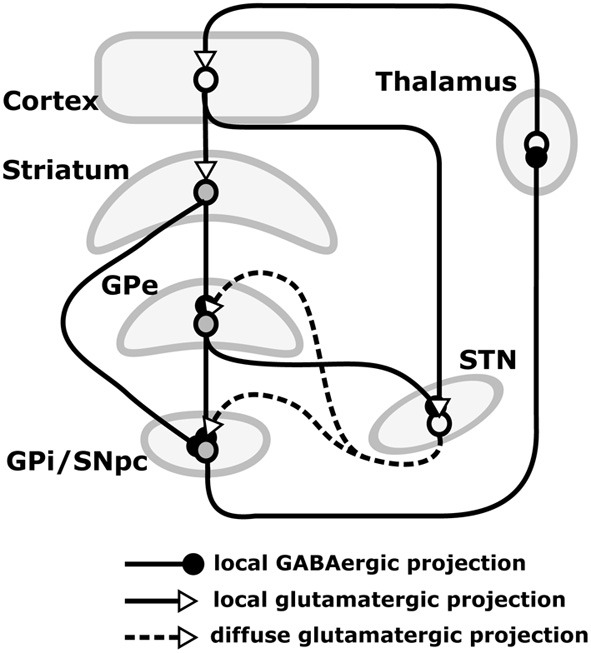
**Internal anatomy of a circuit loop through basal ganglia and cortex**. GPe: globus-pallidus, external compartment; GPi: globus-pallidus, internal compartment; SNpr: substantia nigra pars reticulata; STN: sub-thalamic nucleus.

Our hypothesis must be consistent with the functional implications of these lesions, and so we now review the empirical evidence needed to make sense of them. The basal ganglia (BG) plays a variety of roles in the acquisition and expression of goal-directed behavior, with different territories of BG supporting this diversity of functions. There is a wide agreement that one major function of BG is *selection* (Alexander et al., 1986; Redgrave et al., 1999). The functional anatomy of BG reveals an organization supporting parallel, segregated loops through cortex whose internal structure is substantially invariant (Figure 3; this pattern is, however, different for NAcc shells – NAccSh – see below). Each loop receives the greatest part of its input from a specific cortical region and projects to the same cortical region via the thalamus. *Within each loop*, a cortical cell assembly associated with a particular action or another cortical content excites a focussed part of striatum. This causes inhibition of a corresponding part of the output nuclei of BG (globus pallidum pars interna—GPi, and substantia nigra pars reticulata—SNpr) which, in turn, disinhibits a restricted portion of the thalamus and the related cortex (Chevalier and Deniau, 1990). Thus, within each loop, multiple functional *channels* can select different cortical neural assemblies associated with action representations or other cortical contents (Mink, 1996; Redgrave et al., 1999).

**Figure 4:**
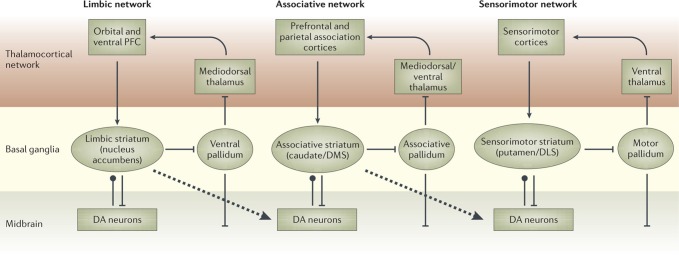
**The three main striato-cortical loops involving different territories of BG and cortical areas**. Reprinted by permission from Macmillan Publishers Ltd: Nature Reviews Neuroscience (Yin and Knowlton, 2006), copyright 2006.

There is now a wide agreement that the functional role of the different BG loops is determined by the contents of the cortical regions they target (Alexander et al., 1986; Romanelli et al., 2005; Yin and Knowlton, 2006). In this respect, the literature often focusses on three main striato-cortical loops also relevant for our hypothesis (Figure 4); note that throughout the paper we use “striato-cortical loop” as short form for the more complete “striato-pallidal/nigral-thalamo-cortical-striatal loop.” The first *sensorimotor loop*, involving DLS, premotor cortex (PMC), and primary motor cortex (M1), is involved in the selection of motor actions based on sensory and motor information. Functionally, this loop plays a key role in the acquisition and expression of habitual instrumental behavior (i.e., the S-R association of Figure 1, Packard and Knowlton, 2002; Featherstone and McDonald, 2004; Yin et al., 2004.

The second *associative loop* involves the DMS (in rats; homologous to caudate in primates) and various associative cortical areas (e.g., inferotemporal cortex—ITC; Middleton and Strick, 1996), parietal cortex (PC; Cheatwood et al., 2003), and also some regions of PFC like PL and the frontal eye fields (FEF; Room et al., 1985; Alexander et al., 1986). Functionally, this loop is involved in orientation, attention, affordance processing, and working memory, all functions related to the cortical regions involved by this loop (Burnod et al., 1999; Hikosaka et al., 2000; O'Reilly and Frank, 2006; Cisek, 2007). Given this role, the loop has been involved in learning and storing the relations between actions and outcomes (Cheatwood et al., 2003; Yin et al., 2005; Yin and Knowlton, 2006).

In contrast to the other two loops, the function of the *limbic loop* through BG is less clear. In rats, this loop involves the NAcc (ventral striatum) and various associative multimodal cortices, in particular the agranular insular cortex (AIC), PL, and infralimbic cortex (IL). In primates the loop also involves OFC and anterior cingulate cortex (ACC). The loop is involved in the highest cognitive processes related to goal-directed behavior and general executive function (Dalley et al., 2004; Hok et al., 2005; Ragozzino, 2007).

Biobehavioral research has produced a wide range of data based on lesion experiments, reversible inactivations, or dopaminergic depletions targeting the NAcc in behaving animals. These data indicate that NAcc is first implicated in the triggering of several low-level, fixed motor behaviors and Pavlovian conditioned responses. In particular the NAccCo is part of a network encompassing the central nucleus of Amg (CeA) and the ventral tegmental area (VTA). The VTA is one of the two major sources of DA, the other being substantia nigra pars compacta—SNpc. SNpc sends DA mainly to DMS and DLS, while VTA sends DA mainly to NAcc, PFC, Amg, Hip. The Amg-NAccCo-VTA network is the neural substrate of autoshaping, the process through which animals can be conditioned to perform various innate behaviors, such as approaching, directed to stimuli predicting rewards (Parkinson et al., 2000a,b; Cardinal et al., 2002b; see also Mannella et al., 2009, for a computational model). NAcc has also been shown to be the root of two effects of Pavlovian associations on instrumental behaviors, namely instrumental devaluation, described in section 2.1, and Pavlovian-Instrumental Transfer (PIT). In the latter, a conditioned stimulus, which has been previously associated with a reward through a Pavlovian procedure, can facilitate an increase in the execution of a previously acquired instrumental action directed to the same or to a different reward (“specific” or “general” PIT, respectively; Corbit and Balleine, 2005, 2011). The NAcc also underlies the role of DA in incentive salience; that is, the motivation to pursue rewards and sustain efforts to accomplish them (Salamone et al., 2003; Niv et al., 2007), processes related to “wanting” (Wyvell and Berridge, 2000; Peciña et al., 2006a). NAcc also plays a role in the hedonic perception of taste/rewarding stimuli (“liking”) measured in terms of specific overt behavioral manifestation (Peciña et al., 2006b).

Overall, this evidence suggests that the limbic loop plays a key role in the Pavlovian prediction of, and attribution of value to, environmental outcomes. Notwithstanding the large amount of evidence available on these processes, we still lack a specific proposal on how different types of values are processed and transmitted to goals to support their selection. Our hypothesis contributes to clarify these aspects.

### 2.3. Computational approaches to goal-directed behaviors

In the last decade, the theoretical understanding of habitual and goal-directed behavior has received a tremendous impetus from machine learning theories on reinforcement learning (RL), founded on dynamic programming and optimal control approaches (Bertsekas, 1987; Sutton and Barto, 1998). Since their inception (e.g., Sutton and Barto, 1987), reinforcement learning methods have strongly cross-fertilized with the bio-behavioral research on instrumental and Pavlovian learning (Houk et al., 1995b), and recently they have become the main theoretical framework to investigate decision making processes (e.g., see Montague et al., 2004; Glimcher et al., 2010). Moreover, reinforcement learning algorithms, in particular those going under the banner of temporal difference (TD) learning (Sutton and Barto, 1998), have become the main modeling tool to understand the dynamics of activation of dopaminergic neurons during conditioning experiments (Schultz et al., 1997). These models have also led to an intense effort to identify the specific neural correspondents of the various components of reinforcement learning algorithms (Houk et al., 1995a; Joel et al., 2002; Botvinick et al., 2008). These computational accounts have given key insights into goal-directed learning and behavior and represent touchstones against which we should compare the implications of our hypothesis, so we now review these models in some detail. On this basis, we will argue that our hypothesis has ramifications which go beyond the understanding given by current theoretical models.

**Figure 5:**
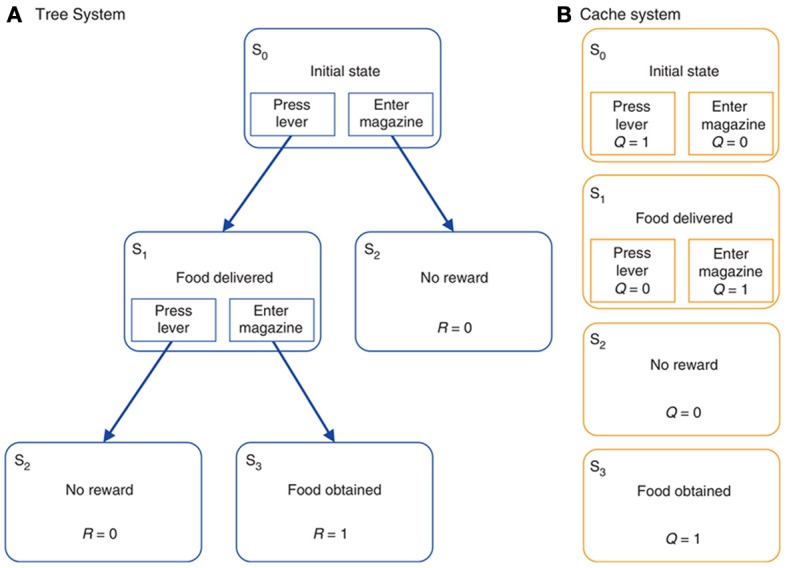
**A typical reinforcement learning task under either (A) goal-directed behavior (“model-based” reinforcement learning) or (B) habitual behavior (“model-free” or “cached” reinforcement learning)**. Reprinted by permission from Macmillan Publishers Ltd: Nature Neuroscience (Daw et al., 2005), copyright 2005.

The model of Daw et al. (2005) (Figure 5) is a useful vehicle to illustrate the links between RL and biology as it also supplies formal definitions of goal-directed and habitual behavior, and thereby explains the relative strengths of these two modes of behavior in different conditions. The starting point of their analysis is the important distinction between *model free reinforcement learning* (MFRL) and *model-based reinforcement learning* (MBRL). MFRL methods, such as the actor-critic model (Sutton and Barto, 1987, 1998), are based on the storing of state values, *V*(*s*), the value assigned to state *s*, and the policy π(*a*, *s*) encoding the probability of executing action *a* given the state *s*. Other MFRL methods, such as *Q*-learning (Watkins and Dayan, 1992), use the value of state-action pairs, *Q*(*s*, *a*), rather than simply states alone. In both cases, “value” is formally defined as the expected sum of future discounted rewards. Value then informs how to generate the action policy either directly, as in the case of *Q*-learning, or indirectly, as in actor-critic methods. Estimates of the values (*V* or *Q*) are updated by the agent acting in the environment on the basis of the experienced reinforcement. MFRL models have been extensively used to capture the processes of acquisition and expression of habit-based behavior (e.g., see Joel et al., 2002; Botvinick et al., 2008).

In MBRL, the agent learns a *forward model* of the dynamics of the environment, formally captured by a *transition function T*(*s*', *a*, *s*) encoding the probability of visiting a new state *s*' when the action *a* is performed in state *s*. The system may additionally model the state-reward contingencies captured by a *reward function R*(*s*) encoding the reward obtained in a given state *s*. In contrast, no such explicit knowledge of the world is available to the agent in MFRL models. The internal representation of a model of the environment allows a MBRL agents to perform more powerful computations than MFRL counterparts. Indeed, using the model, the agent can to some degree evaluate actions internally, thereby making learning more efficient. Second, the transition function *T* is task independent, as it describes the general dynamics of the environment, and stable if the environment is “stationary” (does not change). It may therefore be used to solve multiple tasks captured by different reward functions. For example, if the agent is informed of the change of the structure of rewards it can recompute “on the fly” the values and the policy without the need of re-sampling the environment.

This flexibility of MBRL has however, some costs: due to its computational complexity, MBRL does not scale-up to state/action space domains as large as those that can be dealt with by MFRL. This complexity of MBRL arises from the memory needed to store the transition and reward functions, and the time needed to generate behavior based on the searches of the internal model. Instead, MFRL methods directly “cache” information on policies and so they readily indicate the actions to perform.

The processes of MBRL are proposed to be at the core of goal-directed behavior. In particular, the acquisition of the transition function is analogous to the learning of action-outcome associations postulated in contingency degradation experiments. Moreover, the capacity to reformulate the policy on the basis of internal simulation of the possible consequences of actions when the state-values are updated is analogous to the processes taking place in devaluation experiments, where the change of the value assigned to states (goals) is immediately reflected in different overt behaviors.

Daw et al., (2005) also indicate possible brain systems corresponding to MFRL and MBRL. In particular, the neural substrate of MFRL is the network centered on DLS. This is consistent with experiments showing that lesions of the DLS, or its dopaminergic afferents, prevents animals from becoming habitual even after over-training (Yin et al., 2004; Faure et al., 2005). In contrast, the neural substrate of MBRL is suggested to comprise various cortical (PL, OFC) and sub-cortical regions (DMS, BLA) important for devaluation effects (see section 2.2).

Recently, Solway and Botvinick (2012) have proposed to capture goal-directed behavior processes with probabilistic representations and Bayesian inference. In this way, different processes underlying goal-directed behavior can be isolated as terms in probabilistic expressions and then linked to brain systems implementing analogous functions. In their proposal:

(1) $$\begin{array}{l} p(\pi |s,\hat{r})\propto p(\hat{r}|s,\pi )\cdot p(\pi )=\\ \;\sum_{s^{\prime },a}p(\hat{r}|s^{\prime })\cdot \;p(s^{\prime }|s,a)\cdot p(a|s,\pi )\cdot p(\pi ) \end{array}$$

where $\hat{r}$ and *s*' are respectively future rewards and states, *p*(π|*s*, $\hat{r}$) is the *posterior probability* over the policy given the current state and the rewards, and *p*(π) is the *prior probability* of the policy; other symbols have been defined earlier.

**Figure 6:**
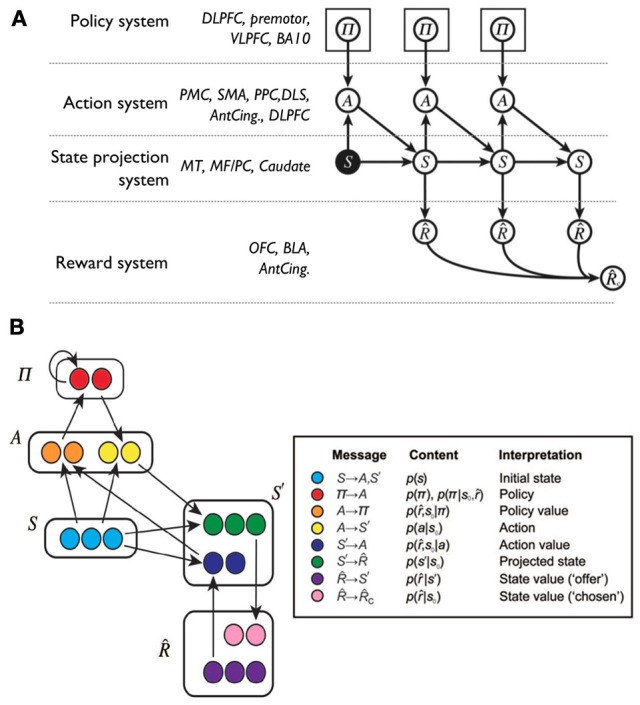
**A Bayesian interpretation of goal-directed learning proposed by Solway and Botvinick (2012). (A)** Graphical model supporting the probabilistic factorization of a model-based reinforcement learning problem, and hence of goal-directed behavior, with a list of possible biological correspondents. ACC, anterior cingulate cortex; BA, Brodmann area; BLA, basolateral amygdala; dlPFC, dorsolateral prefrontal cortex; DLS, dorsolateral striatum; MF/PC, medial frontal/parietal cortex; MT, medial temporal cortex; PFC, prefrontal cortex; PC, parietal cortex; PMC, premotor cortex; SMA, supplementary motor area; vlPFC, ventrolateral prefrontal cortex. **(B)** A possible neural implementation of the functional architecture: based on **(A)** the reader might attempt to link neural areas to the components of the architecture. Adapted and reprinted with permission (Solway and Botvinick, 2012).

The terms in Equation 1 were instantiated in the components of a connectionist model (Figure 6) linked to possible corresponding brain areas. Thus, the prior on the policy *p*(π) is related to the activity of dorsolateral PFC (dlPFC), the policy function *p*(*a*|*s*, π) to motor cortices and DLS, the prediction of states *p*(*s*'|*s*, *a*) to associative cortex and PFC, and the reward expectation *p*($\hat{r}$|*s*') to the activity of the OFC and BLA.

The architecture of Solway and Botvinick (2012) offers a principled overall view of goal-directed behavior but does not account for a key element which is at the heart of our hypothesis, namely the proposal for a key role of the NAcc in the selection of goals within PFC on the basis information on value computed in the limbic brain.

**Figure 7:**
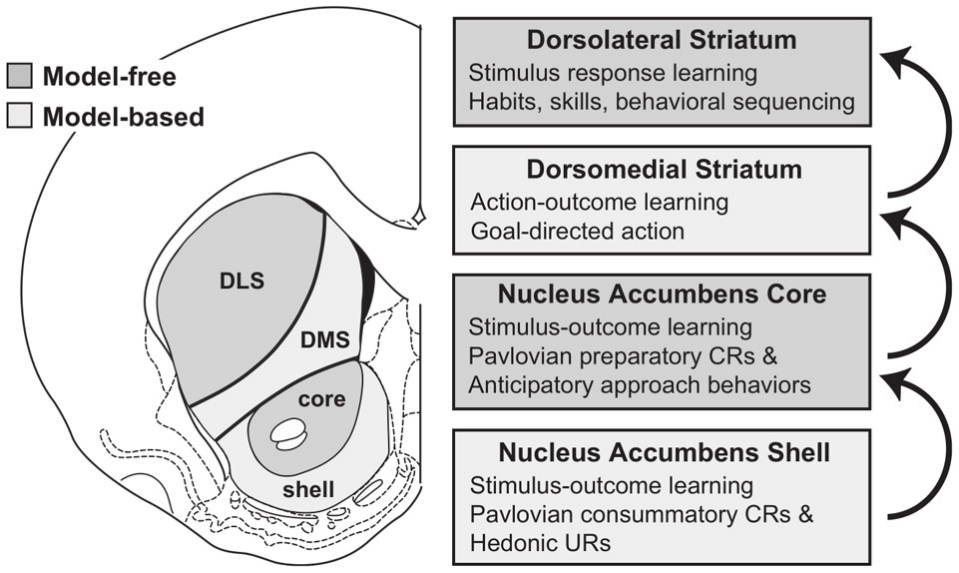
**The proposal of Penner and Mizumori (2011) for the possible functions of nucleus accumbens core and shell, and their relation to downstream striatal regions**. Notice the role of stimulus-outcome predictor ascribed to the accumbens. Reprinted from Penner and Mizumori (2011), Copyright 2011, with permission from Elsevier.

The role of NAcc in goal-directed behavior is also the subject of other computationally-oriented accounts of goal-directed behavior, all referring directly or indirectly to the reinforcement learning framework (Bornstein and Daw, 2011; Penner and Mizumori, 2011; Khamassi and Humphries, 2012). For instance, Penner and Mizumori (2011) (Figure 7) invoke a dual actor/critic framework in which DLS and NAccCo are respectively the actor and the critic of an MFRL system, while the DMS and the NAccSh are the actor and the critic of an MBRL system.

**Figure 8:**
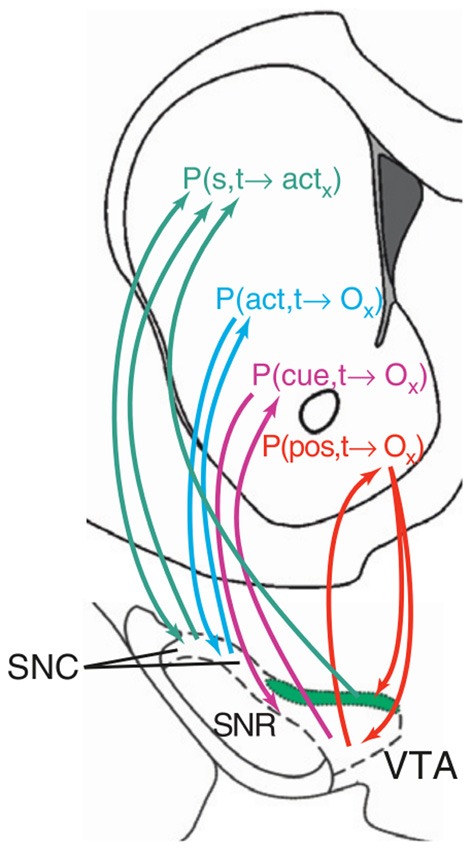
**An hypothesis of ventral striatum as the locus of various types of action-outcome anticipations**. Reprinted from Pennartz et al., (2011), Copyright 2011, with permission from Elsevier.

In contrast, Pennartz et al. (2011) suggest that the actor-critic schema is not the best interpretation of NAcc function (see Figure 8). In their view, different striatal regions compute predictions on outcomes (or actions) based on different types of information. Thus, NAccSh predicts outcomes on the basis of spatial features (e.g., position in space of a certain food resource in a navigation task). Instead, NAccCo predicts outcomes based on specific cues (e.g., visible landmarks). The DMS predicts outcomes based on actions (e.g., the effects of turning right). Finally, the DLS “predicts” the motor actions, considered as lower-level abstractions of outcomes.

The computationally grounded ideas described above clearly represent a major contribution to our understanding of goal-directed behavior. However, they either overlook the critical aspect of how goal selection is linked to value, or they diverge in the way they account for it, so highlighting the need for further clarifications of this issue grounded on available empirical evidence.

## 3. The ventral striato-cortical loop and goal selection

### 3.1. A system-level evolutionary framework for the hypothesis

This section proposes a framework within which we develop our hypothesis on how the NAcc assigns motivational value to goals and thereby participates in their selection. Such a hypothesis is then fully expanded in the remaining sections. We posit that the system directed to accomplish useful outcomes in higher mammals results from an evolutionary trajectory involving three successive “versions” of it having an increasing computational sophistication and power. The additional components of the more recent versions do not replace those of their predecessors, rather they work with them to produce augmented functionality.

**Figure 9:**
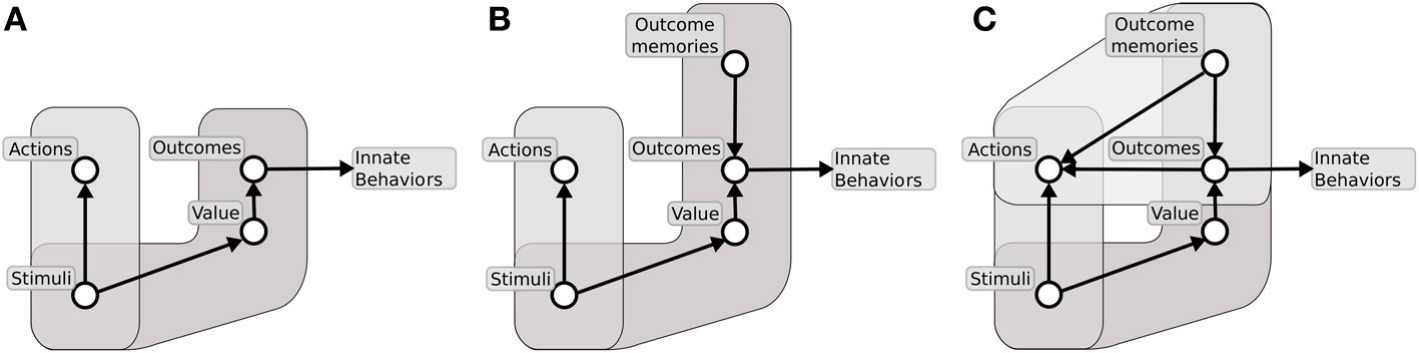
**The three major systems for learning to select desired outcomes forming an evolutionary lineage used here as a background for our hypothesis. (A)** First system formed by instrumental stimulus-response behaviors and simple Pavlovian processes. **(B)** Second system formed by instrumental stimulus-response behaviors and sophisticated Pavlovian processes supported by dynamical neural processes capable of sustained active representations of outcomes. **(C)** Third system formed by instrumental stimulus-response behaviors, sophisticated Pavlovian processes, and further structures allowing outcome representations to recall actions.

The evolutionarily first system (Figure 9A) is formed by two major components: (1) a component capable of learning instrumental habitual behaviors by trial-and-error; (2) a second one capable of forming Pavlovian stimulus-outcome associations. The neural substrate of the first component is mainly a sensorimotor system involving BG motor regions. The substrate of the second component is mainly formed by a network composed of amygdala, ventral BG and other sub-cortical structures (e.g., the hypothalamus and the periacqueductal gray) capable of expressing behaviors which are innate, or the result of early-development, triggered by Pavlovian processes (Davis and Whalen, 2001; Medina et al., 2002; Balleine and Killcross, 2006; Mirolli et al., 2010). The system under discussion is common to all vertebrates (including fish, amphibians, and reptiles), and serves the acquisition of simple behaviors through trial-and-error, the triggering of innate behaviors such as feeding, approaching, avoidance, and orienting, and the implementation of simple Pavlovian processes such as those studied in *delay conditioning* paradigms. None of these behaviors require the maintenance of lengthy memory traces between the conditioned and the unconditioned stimuli (Davidson and Richardson, 1970). Within this system, the ventral regions of striatum mainly support the energization and expression of innate behaviors via its connections to lower motor centers.

The second system to emerge (Figure 9B) builds on the first system, and develops the second component (2) to make it capable of generating more complex Pavlovian stimulus-outcome associations. A major contribution to this empowerment, pivoting on a fully evolved hippocampus, is the implementation of dynamical circuits capable of storing information on stimuli experienced in the recent past. This supports Pavlovian processes taking place in the Amg, and thereby allows the solution of more challenging tasks, such as those involved in *trace conditioning* paradigms. This enhanced Pavlovian system is possessed by more evolved vertebrates, e.g., birds, (Lucas et al., 1981), and allows them to complete more complex tasks where incentive value has to be transferred between temporally distal stimuli (Richmond and Colombo, 2002). This allows them to form conditioned (“secondary”) reinforcers quite distant from actual rewards and capable of driving the acquisition of sophisticated habit-like behaviors. In the new enhanced system, the ventral striatum continues to mainly play a role of energization of action and triggering of innate behaviors, functions still present in mammals and the third system that we now consider (Cardinal et al., 2002b; Gruber and McDonald, 2012).

The third, and evolutionarily most recent, system (Figure 9C) uses the component (1) of its predecessors, has an enhanced component (2), and acquires a third component (3). These enhancements pivot on a fully evolved neo-cortex. The enhancement of component (2) relies on cortical areas such as the AIC and the OFC, dealing with olfaction and taste, and on prefrontal-hippocampal re-entrant connections. These allow the component to have a further enhanced capacity to represent outcomes for long times with respect to Amg alone (Schoenbaum et al., 1998). The third component (3), fully developed in mammals and in particular in primates, is supported by re-entrant circuits involving ventral BG, medial and dorsal prefrontal cortex, and hippocampus, and resulting in a powerful working memory capable of representing experienced stimuli for prolonged periods of time (up to few seconds) (Rolls, 2000a; Euston et al., 2012). The component allows the formation of associations between multiple stimuli in time, and in particular to anticipate future stimuli and outcomes on the basis of the current experience (Funahashi, 2001; Dalley et al., 2004; Matsumoto and Tanaka, 2004). The overall system has an organization and implements the functions analyzed in detail in the following sections.

### 3.2. Outline of the hypothesis

**Figure 10:**
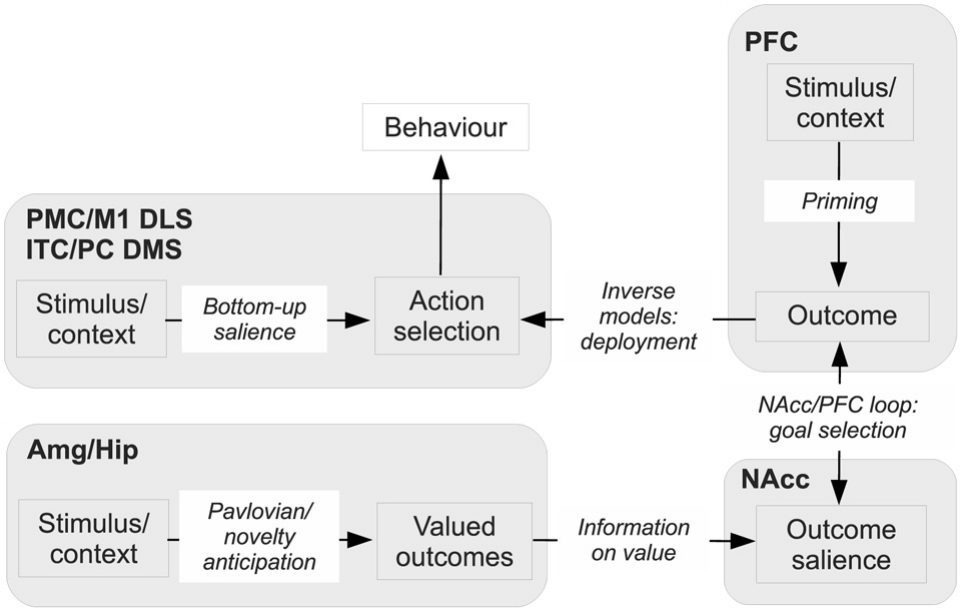
**Sketch of the main functional elements of the hypothesis, with their possible biological correspondents**. Amg, amygdala; DLS, dorsolateral striatum; DMS, dorsomedial striatum; Hip, hippocampus; ITC, inferotemporal cortex; M1, primary motor cortex; NAcc, nucleus accumbens; PC, parietal cortex; PFC, prefrontal cortex; PMC, premotor cortex.

This sub-section outlines the core hypothesis proposed in the paper. The main features of the hypothesis are shown in Figure 10. We will continue to refer back to this figure throughout the rest of the paper as more detail is included in our scheme. The next four sub-sections expand the biological and behavioral evidence supporting the hypothesis in relation to the main brain systems involved (Amg, Hip, NAcc, and PFC).

#### 3.2.1. Amygdala

The Amg encodes unconditioned stimuli (US); that is, appetitive and aversive primary rewarding stimuli. Appetitive stimuli include, for example, food (e.g., its smell and taste), while aversive stimuli comprise objects (e.g., predators) causing body damage and pain. The Amg is one of the limbic brain systems interfacing other brain areas to the homeostatic regulatory systems of the body. On this basis the Amg can modulate the activation of its representation of primary *appetitive/aversive* stimuli depending on the internal state of the animal. For example, if the animal ingests a food, its representation within the Amg can have different activities, hence value, depending on the level of hunger for such food. The activation of USs in Amg can trigger a large number of unconditioned responses (e.g., startle, approach, avoidance), and participates in a number of internal regulatory functions of the body (e.g., heart-rate, salivation) and the brain (via the main neuromodulatory systems). These reactions are made possible by its diffuse projections to multiple subcortical areas and to the NAcc.

The Amg, in particular CeA, implements core Pavlovian processes through which it links representations of CS to innate, unconditioned responses (URs). Moreover, the BLA can associate the representations of CSs to those of USs. This powerful mechanism allows it to associate, in “one shot,” previously neutral stimuli with all the URs associated with any US following the CS. Importantly, this implies that, since the responses are mediated by the CS-US-UR causal chain, the BLA can also regulate the responses on the basis of the *current* internal value assigned to the US (see above).

#### 3.2.2. Hippocampus

Hip is traditionally thought to play a key role in rapid formation of episodic memories and spatial maps for navigation. These memory-based processes rest on the important capacity of the Hip system to detect the novelty of stimuli, of stimuli associations, and of stimuli-context associations. Upon detection of novelty, the Hip is able to activate dopaminergic systems via its projections to NAcc, thereby supporting learning of structures targeted by DA including the Hip itself. This capacity to detect novelty also plays a second function, fundamental to our hypothesis on the attribution of value to goals: information on novelty supplied by Hip to NAcc can also be used to select goals. Indeed, aside from the appetitive/aversive value communicated to NAcc by the Amg, the *novelty of a stimulus represents a fundamental component of the motivational value associated to it*. This because novelty has a pivotal adaptive valence since novel objects, associations, and contexts might have potential appetitive/aversive valence initially unknown to the animal and this can be discovered only by targeting them with the needed attentional, exploratory, and learning resources.

#### 3.2.3. Nucleus accumbens

The NAcc is a nexus for combining stimulus value computed in the Amg and Hip, and for implementing the process of selection of outcomes in synergy with PFC. Thus, NAcc receives information from Amg and Hip which represents the appetitive, aversive, or novelty value of outcomes. At the same time, based on external stimuli, working memory, and internal plans, PFC partially activates or *primes* its internal representations of attainable outcomes offered by the environment. PFC is part of the BG loop with NAcc, and this loop can mediate the selection of PFC outcome representations in the normal way via disinhibition of thalamo-cortical targets. The key, additional mechanism considered here is that NAcc uses the information on value from Amg and Hip to strongly *bias* the selection of specific goals among the multiple, partially activated outcomes encoded in PFC. The fact that NAcc activity is also based on value implies that goals with high value are selected.

The process of selection of goals in PFC is supported in two ways via the two main sub-components of NAcc, namely NAccCo and NAccSh. The NAccCo has the typical structure of the striato-pallidal-thalamo-cortical pathways; it is therefore NAccCo which mediates goal selection in PFC using the “canonical” basal-ganglia selection process described above. Instead, NAccSh contributes to the selection of goals in a different but complementary way, relying on the excitation of DA neurons. Both NAccCo and NaccSh project directly and indirectly to midbrain DA systems (respectively to SNpc and VTA) but the details of the circuits are different. In this respect, we will show that likely NAccSh acts in goal selection by exciting DA that in turns acts at NAccSh, PFC, and other targets including Hip and Amg. This dopaminergic action facilitates selection so causing a rapid switching between candidate goals (thereby promoting exploration of different goals when the animal learns to solve new problems), allows the selection of multiple goals (e.g., goals and sub-goals forming whole behavioral programmes), and facilitates the summation of value from different sources (e.g., related to appetitive/novelty value and to multiple cues and stimuli as in PIT).

#### 3.2.4. Prefrontal cortex

As mentioned above, PFC forms a striato-cortical loop with NAcc. It is possible to distinguish three sub-systems within this loop, each performing a different function related to the anticipation of action-outcomes and the encoding of goals. The first sub-system, based on NAcc/AIC connections (in rats; in primates, also NAcc/OFC connections), contributes to select “ultimate” (distal) biologically-valuable outcomes, for example “food ingestion.” These goals are encoded in AIC and OFC in terms of their features most closely related to their appetitive aspects, in particular odor and smell. The second sub-system, based on NAccCo-PL connections, contributes to select outcomes based on their more cognitive aspects, such as their visual and auditive aspects. This system might be particularly important for encoding goals based on novelty. In primates, this system is also corroborated by the connections between PL and dlPFC, encoding not only ultimate goals but also proximal/sub-goals instrumental for the achievement of ultimate goals and initially not characterized by an intrinsic biological valence. The third system, mainly based on NAccSh-IL connections (also NAccCo-IL in primates), plays the role of avoiding the selection of Pavlovian and instrumental behaviors which are either no longer useful or even detrimental. The PFC also exchanges multiple direct and indirect connections with motor areas and modal sensory associative areas (e.g., the ITC and the PC) and uses these connections to implement sophisticated forward and inverse models that allow it to trigger the execution of suitable actions directed to pursue the selected goals.

#### 3.2.5. The functioning of the whole system

We now present an example of how the whole system works referring to Figure 10. This example gives a first intuition of how the whole hypothesis works, while several aspects of the functioning of the various components, and the empirical evidence supporting them, are explained in detail in the following sub-sections. In the example, Amg uses the current perceived state of the world, or “input stimulus” (e.g., the sight of a lever) to activate an US associated to it (e.g., the valuable aspects of food, such as its taste and odor). We also imagine that the outcome has some novel aspects (e.g., imagine a food cooked in a novel fashion): this implies that its representation is strongly active in Hip and this contributes to increase its value. In prefrontal cortex (PFC) the same input stimulus (lever) primes a perceptually more sophisticated representation of the food outcome (e.g., not only taste and odor, but also sound/visual appearance of the food). Information on possible outcomes (PFC), on their appetitive/aversive value (Amg), and on their novelty (Hip), is integrated in accumbens where it forms their current *saliency*. By “saliency” we mean the overall activation of an internal representation, based on different sources of information encoding the current biological relevance of the represented item for the organism.

The entire process is supported by DA caused by NAcc-VTA/SNpc and reaching the various components of the system. Based on saliency, the NAcc-PFC loop selects the outcome in PFC having the highest saliency, so designating the goal that will drive action selection. In parallel, the input stimulus also primes the bottom-up activation of different actions within the DLS-PMC/M1 loop, but none gets enough activation to be triggered (e.g., assuming that habits are still not fully formed). However, the goal now selected in PFC leads to produce a top-down bias on the DLS-PMC-M1 loop that leads to select and perform the action that allows its accomplishment.

### 3.3. The amygdala: appetitive motivations

**Figure 11:**
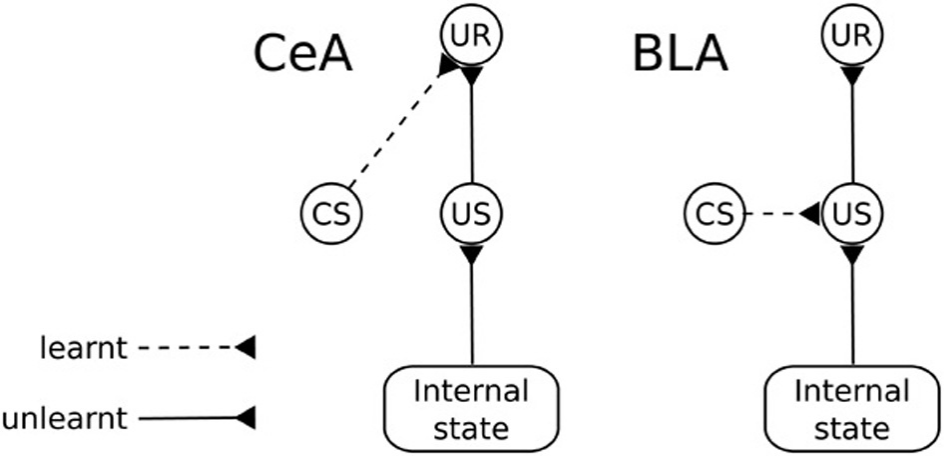
**Functional differences between the basolateral amygdala (BLA) and the central nucleus of amygdala (CeA)**. CS, conditioned stimulus; US, unconditioned stimulus; UR, unconditioned response.

The amygdala (Amg) is formed by a group of nuclei acting as a central hub for the processing of appetitive and aversive motivational information. An important function of Amg is to trigger unconditioned behavioral responses (UR; e.g., orienting, startle, approaching) and to regulate a number of bodily processes (e.g., blood pressure, heart rate, salivation), following the perception of unconditioned stimuli (Davis and Whalen, 2001). These “primitive” responses are triggered via projections to sub-cortical structures (Behbehani, 1995; Bandler et al., 2000; Davis and Whalen, 2001; Balleine and Killcross, 2006). An important aspect of these responses is that Amg is capable of regulating their triggering “on the fly” based on the current state of the body (Hatfield et al., 1996). For example, the reactions of approach and salivation in response to a foodstuff might be inhibited if the animal has been previously satiated by that foodstuff. Amg also plays a key role in Pavlovian processes (Medina et al., 2002; Balleine and Killcross, 2006). When an animal experiences a neutral stimulus in a stable temporal relation with an “unconditioned stimulus” (US; i.e., an unlearned motivationally salient stimulus), Amg is capable of forming Pavlovian associations between them so that the neutral stimulus becomes a conditioned stimulus (CS). Such Pavlovian associations can be stimulus-specific or response-specific (Balleine and Killcross, 2006). wo different groups of nuclei within the Amg are responsible for the two kinds of associations, respectively the BLA and the CeA (Figure 11).

Lesion and inactivation studies of the BLA reveal that the CeA supports Pavlovian conditioning in a “US-dissociated” response-specific way, i.e., it fails to produce the behaviors typical of devaluation experiments. In particular, after BLA lesion (revealing the functioning of CeA in isolation), the animal learns to associate the CS with the same unconditioned responses (UR) that were associated with the US irrespective of the current value of the US. For example, a rat responds to a light consistently associated to food even if the animal has been satiated for that food (Hatfield et al., 1996).

In contrast, the BLA can associate a UR to the US representation so that the Pavlovian response associated with a CS remains tied to the representation of the US that caused the association (Hatfield et al., 1996). This process is based on the formation of links between the neural representations of the CS and US so that the presence of the CS recalls the internal representation of the US, including its current motivational value. Thus, changes in the reward value of the US results in changing the ability of the CS to recall the Pavlovian responses (Balleine and Killcross, 2006; Mirolli et al., 2010). The BLA, which can be considered an evolutionarily later, more sophisticated addition to the Amg complex, can exert important control functions on the activity of the CeA. In particular, it can affect its activation based on the current motivational value of stimuli in conditions where CeA alone would be insensitive to this, for example in the case mentioned above related to the responses to a CS linked to a devalued outcome (Balleine and Killcross, 2006; Mirolli et al., 2010).

Important for our hypothesis, CeA influences NAcc through the modulation of VTA dopaminergic neurons (Fudge and Haber, 2000; Fudge and Emiliano, 2003). Both Pavlovian autoshaping and general Pavlovian instrumental transfer depend on CeA, NAcc, and VTA (see section 2.2 and Corbit et al., 2007). Interestingly, the influence of the CeA over the NAcc results in US-dissociated effects as described above (Mannella et al., 2009).

The BLA sends to the NAcc one of the major afferent projection streams received by this area. As noted in section 2.2, the information conveyed through this pathway is necessary for the learning and expression of instrumental devaluation (Balleine et al., 2003). In general, the BLA conveys to NAcc information about USs and about USs predicted by CSs. Important for our goal-selection hypothesis, the level of activation of the representations of USs (i.e., outcomes) in BLA, which is modulated by the internal state of the animal as illustrated above and is communicated to NAcc, encode the value that Amg assigns to them.

### 3.4. The hippocampus: novelty and the motivation to explore

Another major source of projections to NAcc, especially to NAccSh, is the Hip; Voorn et al., 2004; Humphries and Prescott, 2010). The hippocampal complex comprises several areas characterized by distinct neural organization and computational mechanisms (Rolls and Treves, 1998). Among the most prominent, the enthorinal cortex (EC), relaying information from associative cortical areas (mainly PFC, PC, and ITC) to the dentate gyrus (DG) in Hip, performing recoding of its input in sparser form (thus enabling orthogonality); the CA3 layer of Hip, performing auto-associative fast memory encoding based on its multiple re-entrant connections; and CA1 layer of Hip, recoding information from the hippocampal system before relaying it (via the subiculum—Sub, and EC) back to the cortical areas projecting to Hip.

There is currently a lively debate on the nature of the information reaching NAcc from Hip which centers on two main theories: one related to the role of Hip for episodic memory and one to its role in spatial cognition and navigation (see Pennartz et al., 2011 for a review).

Hip plays a pivotal function for the fast, possibly one-shot, acquisition of integrative memories of “episodes”—specific, contextualized experiences (Eichenbaum et al., 1999; Eichenbaum et al., 2004; Smith and Mizumori, 2006; Bird and Burgess, 2008). In this respect, space is only one of several dimensions of the information stored by Hip. The stored memories last for hours or days (Rolls and Treves, 1998), and are supported by long term potentiation (Frey and Morris, 1998). The memories so formed are eventually consolidated within most of the cortical mantle with which the Hip shares important re-entrant connections (McClelland et al., 1995).

Given these properties, in particular its capacity to quickly store information about integrated context, Hip also plays a key role in spatial navigation. This is indeed one of the first and most studied functions of Hip (O'Keefe and Nadel, 1978; Mulder et al., 2004; Kumaran and Maguire, 2005). In this respect, evidence shows that Hip can form “spatial maps”—allocentric representations of space that allow animals to self-localize and navigate in space (O'Keefe and Burgess, 1996). This research has also led to several computational models of how hippocampal projections to NAcc support path integration and spatial planning. The Hip also plays a key function in decision making in goal-directed navigation tasks. For example, it has been shown that, at decision points in a maze, the rat Hip performs “mental simulations” of the possible alternative courses of actions and NAcc evaluates the outcomes (Johnson et al., 2007; Johnson and Redish, 2007; see Baldassarre, 2002, and Pezzulo et al., 2013, for some models).

In this paper, we propose a third key function of Hip-NAcc projections, but which is closely related to the role of Hip in episodic memory. Thus, we hypothesise that Hip-NAcc projections communicate *novelty-related value* to NAcc. The literature on novelty detection in Hip is large (see below) and much of it considers novelty detection as a process supporting the formation of episodic memories. The new aspect of our hypothesis is that we propose that hippocampal novelty detection also serves a second important function: the assignment of value to goals. Our proposal is therefore not at odds with the extensive evidence showing Hip mediating episodic memory; rather, it *adds* to this previously proposed function by highlighting the unifying function of novelty detection—for episodic memory or value assignment. To articulate this further, we now first review the relevance of the Hip novelty-detection capacity for memory formation, and then we expand the idea of how novelty value supports goal selection.

Novelty detection can be seen as a process required for the formation of episodic memory. An animal is continually bombarded by a large amount of sensory information, and so the detection of novelty allows filtering of stimuli and events that deserve engagement of learning processes. To this purpose, the hippocampal system and surrounding areas (e.g., the perirhinal cortex) are capable of detecting various forms of novelty, from *stimulus novelty* to *associative novelty* and *contextual novelty*: the literature on these topics is now very large (see Ranganath and Rainer, 2003, and Kumaran and Maguire, 2007, and for two excellent reviews).

**Figure 12:**
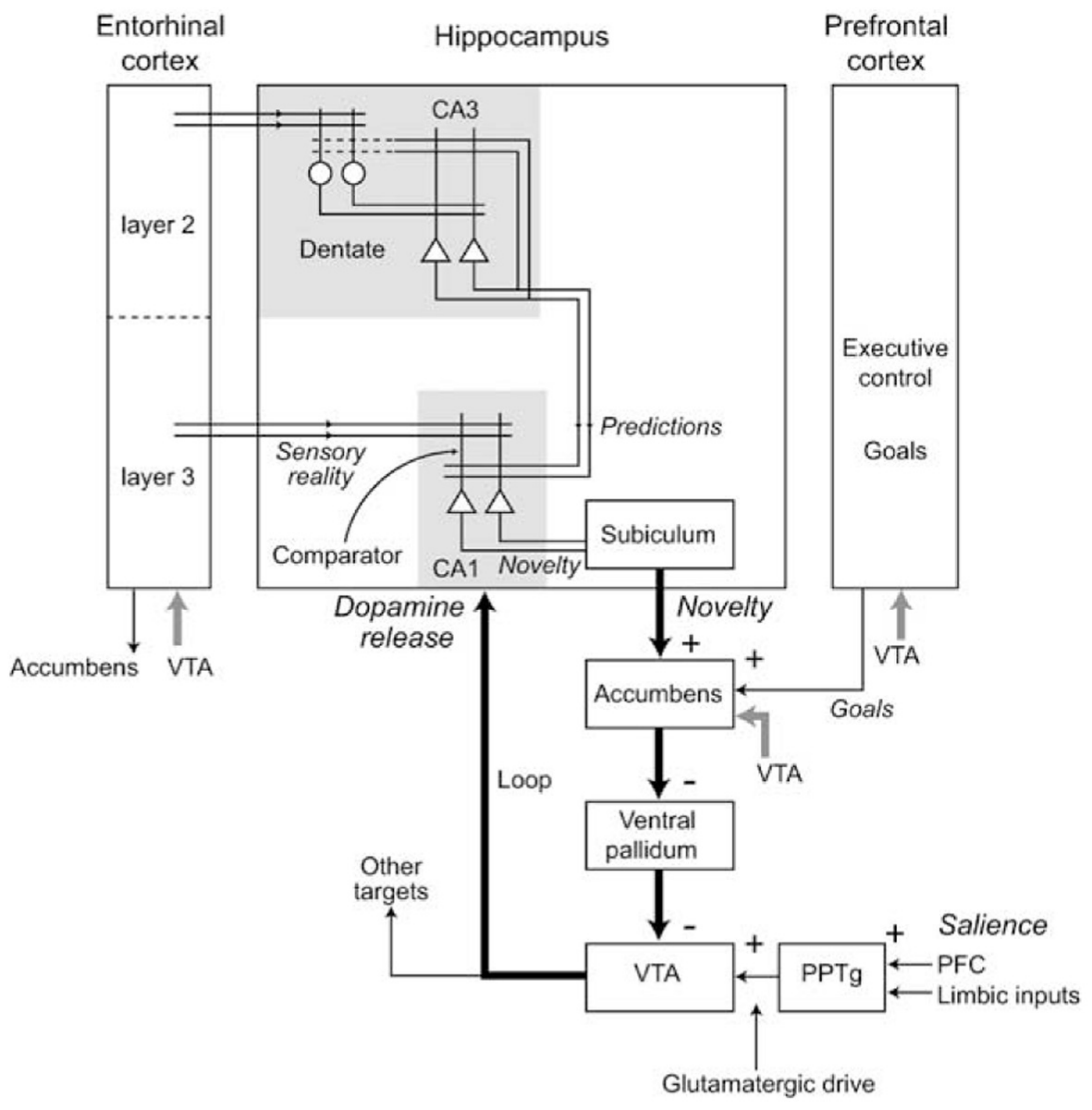
**Various components of the Hippocampal system underlying novelty detection in Hip, and the consequent production of dopamine via indirect connections to the VTA**. Reprinted from Lisman and Grace (2005), Copyright 2005, with permission from Elsevier.

Novelty detection in Hip might be implemented by a process that compares the actual experience with the Hip predictions or memories, detecting the mismatch between them (Hasselmo et al., 1995; Lisman and Otmakhova, 2001; Meeter et al., 2004; Karlsson and Frank, 2008; Van Elzakker et al., 2008). In particular (Figure 12), it has been proposed that CA1, receiving input from both EC and CA3, might compare the memories recalled by CA3 and the “reality” received from EC, and might detect the novelty of stimuli on the basis of the mismatch between them. The novelty of an experienced stimulus/event/context does not decay with a single experience but lasts for the time needed for it to be explored and memorized (i.e., to become “familiar”).

The Hip is also capable of responding to cues which predict novel stimuli—so-called “novelty anticipation” (Wittmann et al., 2007). In this case, stimuli that predict the arrival of novel patterns (e.g., images) activate Hip more strongly and also cause the dopaminergic system to fire, similarly to what happens with the anticipation of appetitive rewards. This process might be important for the assignment of novelty value to cues anticipating novel outcomes.

We now have the information needed for explaining our proposal on the role of Hip in assigning novelty-based value information to goals via NAcc. The idea is that Hip projections to NAcc have an effect that goes well beyond the indirect modulation of it via VTA DA signals. In particular, such projections are fundamental for informing NAcc of stimuli/outcomes/contexts which have a high novelty-based motivational value and this information is used by NAcc to select goals related to them. In this way, novel stimuli/outcomes/contexts become the focus of attention and exploratory activities are directed at interacting with them. This, in turn, facilitates the agent's understanding of the environmental processes producing the novel stimuli and of any role the agent might have in their causation. The adaptive utility of this is that novel objects and contexts have a high biological valence since they represent potentially important threats or opportunities. The biological importance of novelty is clearly shown by the fact that, when set in a novel environment, hungry animals prefer to explore the environment before eating available food, and by the close relation between novelty and fear-related processes (Cavigelli and McClintock, 2003). Brain imaging evidence shows a strong relation between the DA-related processes driving exploration based on novelty and the consequent possible achievement of rewards (Bunzeck et al., 2012). Having selected a goal based on its novelty (via the Hip-NAcc-PFC circuit) the accompanying release of DA in other brain areas (via Hip-NAcc-VTA) promotes the required learning of memories related to it (Lisman et al., 2011) and agent-environment interactions responsible for the novelty.

The novelty detection process of Hip also strongly interacts with DA production via NAcc. In this respect, Lisman and Grace (2005) have proposed an important theory for which Hip novelty detection modulates the activity of dopaminergic neurons of VTA via an indirect pathway involving Sub and NAcc (see Figure 12). According to this hypothesis, novelty detection in Hip would activate dopaminergic areas projecting back to Hip (aside several other cortical and sub-cortical areas) thereby supporting the formation of memories. Although DA projections to Hip are rather sparse (Gasbarri et al., 1997), the DA injected in Hip might nevertheless mediate plasticity to support the memorization of novel stimuli (Otmakova et al., 2013). In accord with the idea of DA influencing the formation of memories, it has been shown that hippocampal input to NAccSh is needed for the expression of the *latent inhibition effect* (Peterschmitt et al., 2005; Meyer and Louilot, 2011; Quintero et al., 2011). This effect occurs when Pavlovian conditioning is substantially slower if the CS has been previously become familiar for the animal in absence of any reward (Lubow and Moore, 1959). This is consistent with the idea that a novel CS detected by Hip causes a release of DA by activating VTA via NAcc, and this in turn enhances Pavlovian learning.

### 3.5. The ventral striatum: an integrator of value for goal selection

Within the proposed hypothesis, ventral striatum is supposed to act as a nexus for integrating stimulus value from Amg and Hip, and using it to bias goal selection in PFC. Amg and Hip are also *directly connected* to PFC. These connections are important for the basic functions explained with respect to the evolutionary perspective presented in section 3.1. We now analyze them as this also allows a better clarification of the different role played by the connections between those areas when they are mediated by the NAcc.

#### 3.5.1. The function of the direct connections between amygdala/hippocampus and prefrontal cortex

The direct connections between Amg and PFC are first of all a means to enhance Amg-based Pavlovian learning processes via the working memory capabilities of PFC. This might have been an important evolutionary step leading to strengthen Pavlovian processes (Figure 9B). In particular, Amg, AIC (involved in processing smell and taste) and OFC (also involved in smell and taste processing) operate as an integrated system with OFC showing patterns of neural activity similar to those of Amg but more robust with respect to time delays (e.g., involved in trace conditioning experiments; Runyan et al., 2004) and complex situations (e.g., those involving contextual shifts; Schoenbaum et al., 2003). In section 3.1 we suggested that this system might have been a way to empower Pavlovian processes in Amg, and it might have also been a precursor for the emergence of the more sophisticated functions of PFC in goal management, especially in primates. NAcc plays an important role in these enhanced Pavlovian processes, aside its role in goal-selection illustrated below. In Particular, the NAcc might be an important behavioral output gateway of Pavlovian processes thanks to its connections to sub-cortical structures (e.g., for triggering basic behaviors such as approaching, orienting, etc., Parkinson et al., 2000b; Cardinal et al., 2002b; Gruber and McDonald, 2012).

The strong direct connections between Hip and PFC, instead, allow the Hip-PFC axis to support working memory and planning functions, thereby forming an integrated system supporting the anticipation of possible future states that might follow from the execution of actions in the current state (Fuster, 1997; Frankland and Bontempi, 2005; Bast, 2007). The formation and progressive sophistication of this system has been an important evolutionary step leading to strengthen the general “executive function” of organisms (Figure 9C). The key aspects of the relation between the two systems are that PFC can perform reasoning and planning processes by relying on dynamical mechanisms supporting working memory, while Hip can quickly form broad associations, e.g., involving multimodal stimuli and context. Together, the two mechanisms generate a powerful computational machine for supporting planning, reasoning, and executive functions (Toni et al., 2001; Bast, 2007).

#### 3.5.2. Anatomy and connections of nucleus accumbens core and shell

We now consider some features of NAcc internal anatomy, functioning, and external connectivity important for understanding how different sub-regions of NAcc contribute to select goals in differential ways. As already noted, it is possible to identify at least two subregions within the ventral BG, based on the circuits of NAccCo and NAccSh. These two circuits differ in cytology, micro-architecture, and afferent/efferent connections with other neural regions (Zahm, 2000; Voorn et al., 2004; Humphries and Prescott, 2010).

**Figure 13:**
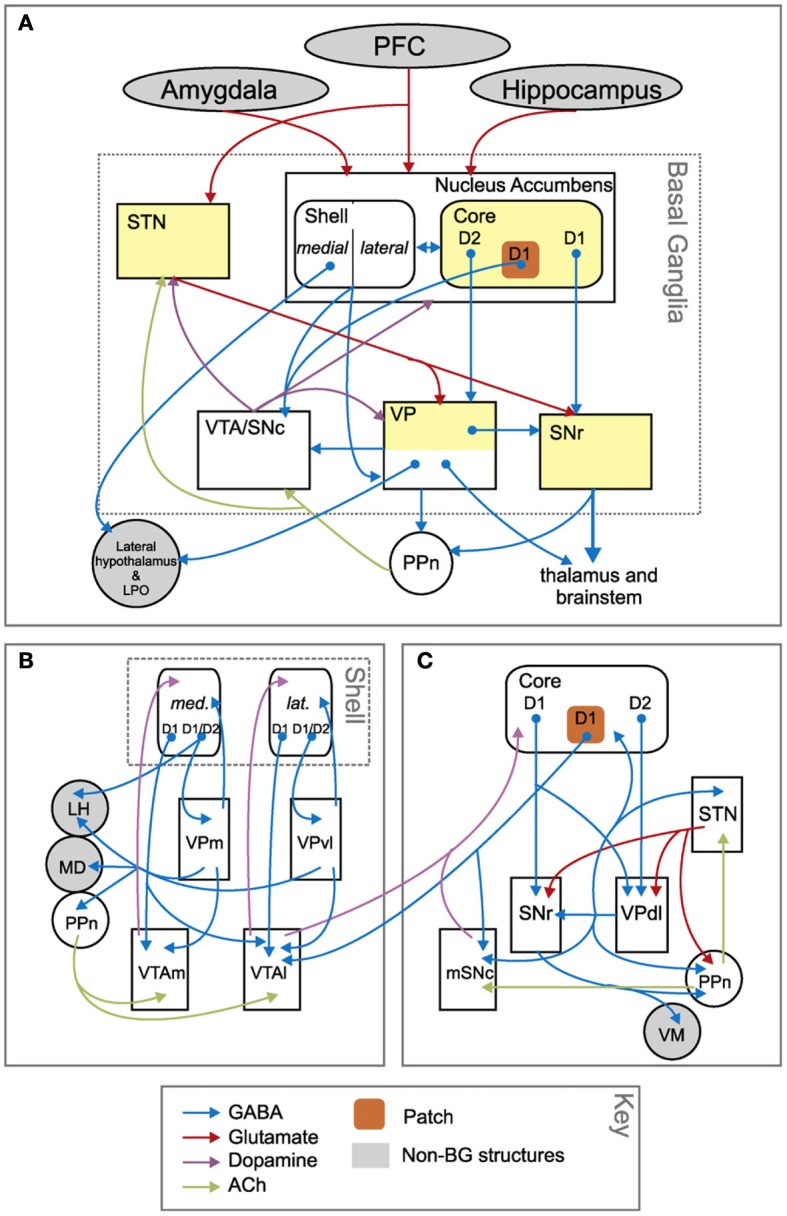
**Anatomical differences between the basal ganglia circuits involving nucleus accumbens core and shell. (A)** Overall schema of the connections involving the whole nucleus accumbens. **(B)** Zoom on the connections involving the nucleus accumbens shell. **(C)** Zoom on the connections involving the nucleus accumbens core. Reprinted from Humphries and Prescott (2010), Copyright 2010, with permission from Elsevier.

The BG-cortical loop involving NAccCo reproduces almost the same cytology and internal organization as the other BG-cortical loops (see Figures 3, 13), so making it ideal for implementing selection processes. In particular, NAccCo is connected to the ventral globus pallidus and SNpr, and the latter projects to thalamus which is in recurrent connectivity with cortex. The ventral globus pallidus and SNpr are also innervated by the subthalamic nucleus (STN). This micro-circuit involving striatum, STN, pallidum, and SNpr, has been closely linked with the capacity of basal-ganglia to perform the selection of the contents of the targeted cortex (Gurney et al., 2001; Humphries and Gurney, 2002). The cortical areas involved in the loops with NAccCo are AIC and PL in rats, and also OFC and ACC in primates.

In contrast, the BG circuit involving NAccSh shows some unique features in terms of both cytology and micro-architecture (see Figure 13). In particular, VP (medial and ventrolateral regions) is the only BG output nucleus of the NAccSh which so has no access to SNpr. Moreover, and importantly, the circuit has no connectivity with STN, so it is mainly formed by the “direct pathway” of BG but lacks the “indirect pathway” involving the STN. The latter feature implies that NAccSh cannot perform a strong “winner-take-all” selection as it cannot fully inhibit the non-selected competitive options in cortex (Humphries and Prescott, 2010; cf. Gurney et al., 2001, on the importance of STN for BG to perform competitive processing).

In terms of connectivity, in rats NaccSh targets the AIC, the PL and the IL. IL plays a role in inhibiting instrumental behaviors and in the extinction of Pavlovian processes (Quirk et al., 2000; Coutureau and Killcross, 2003; Rhodes and Killcross, 2004; Sotres-Bayon and Quirk, 2010). In primates, NAccSh also targets OFC and ACC.

NAccSh and NAccCo also differ in their relation to midbrain DA systems (Voorn et al., 2004; Humphries and Prescott, 2010). Thus, only a subset of NAccCo projection neurons—comprising the so-called “patch”—project to DA neurons in SNpc. DA produced by SNpc mainly targets striatum. In contrast, most projection neurons in NAccSh project to the dopamine neurons in the VTA. DA produced by VTA mainly targets NAcc, Amg, Hip, and PFC. In both NAccSh and NAccCo, the relevant parts of VP project with GABAergic (inhibitory) synapses to their respective DA systems. Critically, in the circuit with NAccSh there is no excitation of VP from STN (see above) and this might lead NAccSh to regulate DA differently with respect to NAccCo, as explained below.

#### 3.5.3. Dopamine and goal selection

Dopamine modulation plays an important role in the goal-selection processes of NAcc, so we now briefly consider the DA processes that might be more relevant for goal-selection, in particular the so called *dopamine transients* happening at a time-scale of seconds. Dopamine can be produced at different times scales by different causes, and plays different functions within the targeted areas. *Tonic* DA release is caused by the removal of inhibitory constraints affecting spontaneously active dopaminergic neurons (Floresco et al., 2003; Floresco, 2007). The resulting DA concentrations within the targeted areas are commonly measured through microdyalisis at a time-scale of minutes and even hours. Tonic DA has a pivotal role in enhancing the performance of motor behavior, as shown by the impairment of motor behavior in Parkinson patients after its decrease in sensorimotor regions of striatum (Redgrave et al., 2010). Related to this, tonic DA has been linked to the disposition to sustain efforts in pursuing goals (Salamone et al., 2003). Tonic DA at very slow time scales has been also related to the experience of stressors (see Cabib and Puglisi-Allegra, 2012, for a review). Based on this type of evidence, tonic DA has been proposed to be regulated on the basis of the obtained average reward and to mediate the vigor with which actions are performed (Niv et al., 2007). *Phasic* DA release results from a direct glutamatergic excitation of dopaminergic neurons having a duration at the time scale of milliseconds. There is a large agreement that phasic DA plays a key role in learning processes. In particular, a main proposal is that phasic DA reflects reward prediction errors (Schultz et al., 1997; Schultz, 2002) and guides trial-and-error learning processes within BG (Houk et al., 1995a). Phasic DA has alternatively been proposed to be caused by sensory prediction errors caused by unexpected phasic events, and on this basis to drive the formation of actions (Redgrave and Gurney, 2006; see Mirolli et al., 2013, for a computational model that reconciles the two proposals).

The recent introduction of fast-scan cyclic voltammetry (FSCV) shows that extrasynaptic DA concentrations can also change at a time-scale of seconds and subseconds (*dopamine transients*; Robinson et al., 2003). One possible cause of DA transients is the spreading of burst firing activity in a large population of dopaminergic neurons in a spontaneous active state (Floresco et al., 2003). Such magnified bursts result in a large dopaminergic efflux that might overflow outside the synapse into the extracellular space. Studies using FSCV show that DA transients are evoked by salient appetitive, aversive, and novel stimuli (see Horvitz, 2000, for a review). For instance, Roitman et al. (2004) show that subsecond DA signaling acts in the NAcc as a real-time modulator of food-seeking behavior. Other studies show that the production of DA transients can be caused by novel stimuli (Rebec et al., 1997; Robinson and Wightman, 2004; Robinson et al., 2011). Here we propose that DA transients might have a relevant role in goal selection as they have the suitable time scale. In particular, they are slow enough to affect selection processes happening within BG (while single phasic DA bursts might be too fast for this). At the same time, they are fast enough to affect the selection of different goals in time (while the dynamics of tonic DA might be too slow for this purpose).

Establishing the specific effects of different DA levels on goal selection is not easy. Dopamine (especially phasic, see above) might indirectly affect the selection of actions by guiding the history of the reinforcement learning processes that result in a certain behavior. Dopamine (especially tonic, see above) might also regulate the overall “selection mode” over long periods of time, e.g., by energizing or depressing decision making in the presence of appetitive or aversive/stressing conditions. The literature is now investigating a more direct causal role of DA levels in decision making, in particular when a choice between different options is requested. For example, Morris et al. (2006) found a close link between DA levels and the choice of different actions leading to a reward with different probabilities. However, they also argued that, due to its diffused and unspecific nature, DA can only reflect decisions once they have been taken elsewhere. Aside the effect of DA on decisions through learning, McClure et al., (2003) have proposed a direct effect of DA levels on the probability of selection of actions reflecting “incentive salience,” or “wanting,” i.e., the motivation to perform the action directed to gain a reward anticipated by a cue (Berridge, 2004, 2007). Below, we propose specific mechanisms through which DA might affects goal-selection processes happening within NAccCo and NAccSh that in part reconcile these positions.

#### 3.5.4. Different role of nucleus accumbens core and shell in PIT

NAccCo and NAccSh play differential roles in Pavlovian to instrumental transfer processes (PIT): these are relevant to understand the differential role they play in value-based goal selection. Section 2.2 already illustrated that there exist two forms of PIT, the general PIT (gPIT) and the outcome-specific PIT (osPIT). NAccCo and NAccSh have dissociated roles in gPIT and osPIT (Corbit et al., 2001, 2007). In particular, evidence based on lesions shows that NAccCo is necessary to have devaluation and gPIT, but not osPIT (Corbit and Balleine, 2011). In contrast, NAccSh is necessary to have osPIT but not devaluation and gPIT. These results are somewhat surprising. Indeed, while the relevance of NAccCo for devaluation seems to indicate its sensitivity to the value of different goals and hence in their selection, its role in gPIT seems to indicate a role in producing general effects of action energization. At the same time, while the lack of relevance of NAccSh for devaluation seems to indicate no role for the differential selection of goals, its role in osPIT seems at odds with this. In what follows, we describe a possible resolution of this apparent impasse.

#### 3.5.5. Role of nucleus accumbens core in goal selection

The evidence presented above allows us to propose an hypothesis on how NAccCo and NAccSh might contribute to select goals within PFC in complementary ways. As mentioned above, NAccCo, which shares a selection circuit with the rest of BG, is able to take part in selecting the contents of the cortex within the striato-cortical loop to which it belongs, namely PFC (Humphries and Gurney, 2002; Gurney et al., 2012). The cortical targets within these loops are at least of two types (Fuster, 1997): (a) the representations of the rewarding aspects of outcomes (e.g., visceral, gustative, olfactive) encoded in AIC and OFC; (b) the representations of more abstract aspect of outcomes (e.g., visual and auditive) received from the outer world and encoded in PFC regions such as PL. All these features of goal representations are the subject of selection supported by NAccCo.

We propose that the PFC contains *partially activated* representations of possible future outcomes *primed by perceived environmental conditions* on the basis of PFC capacity to reason on future states. These patterns of activity project to neurons in NAccCo where they are integrated with the information of value from Amg and Hip to form a measure of overall level of activity or *salience*. Information of value plays a key role in the NAccCo selection as this is also targeted by Amg and Hip inputs (O'Donnell and Grace, 1995; Finch, 1996). In particular, information on the appetitive/aversive value of stimuli received from Amg, and on their novelty value received from Hip, is encoded in NAcc on the basis of the “common currency” of saliency. In this way, Amg and Hip are able to *bias* the selection of outcomes on the basis of value. Salience of outcomes is at the base of the selection that NAccCo performs through the competitive processes also common to the rest of BG (Redgrave et al., 1999) and for which inputs with larger salience are selected (Gurney et al., 2001). The mechanism of selection in one BG loop governed by a biasing input from a source outside the loop is a common theme in some of our quantitative models of BG selection (Lewis et al., 2011; Baldassarre et al., 2012; Shah et al., submitted).

The term “bias” in general connotes a *linear* mixing of salience components and it might be argued that sufficiently strong cortical inputs would allow goal selection that could override any limbic (Hip and Amg), value contribution. However, there is evidence that limbic inputs to NAcc interact non-linearly with their cortical counterparts, and can in fact *gate* or veto these inputs (O'Donnell and Grace, 1995; Goto and O'Donnell, 2002). This mechanism provides ideal support to the value-based guidance of goal selection proposed here. So, even if we will continue to refer to “biasing” of cortical input at the level of NAcc, there will be no presumption of linear control of salience.

The connections from PFC to NAcc might not only contribute to the computation of saliency and goal selection, but also to the formation of the Amg/Hip-NAcc connections that allow Amg and Hip to assign value to goals in the first place. The idea is as follows. When rewarding/novel outcomes are first experienced, their representations get formed and strongly activated in PFC, for example in terms of multimodal visual/auditory features. Information on these outcomes encoded in PFC is projected back to NAcc. At the same time, the representation of the rewarding/novel outcomes are also strongly activated in Amg (e.g., in terms of odor and smell) and/or Hip (e.g., in terms of multimodal aspects of the outcome). These would allow the formation of connections between outcome representations in NAcc and those in Amg and Hip. In later stages, these connections would allow Amg and Hip to contribute to communicate the saliency of outcomes to NAcc so as to bias the selection of specific outcomes primed in PFC (for a model of some of these processes, see Baldassarre et al., 2012).

This view on the role of NAccCo in selecting goals also agrees with its role in devaluation experiments. In these experiments *only one goal has a high value* while the others are devalued. So, for example, in a typical devaluation experiment the sight of two different manipulanda elicits the activation of two possible outcomes in PFC related to the two foods achievable by acting on them. However, Amg is able to inform NAcc of the current value of each of the two outcomes, based on the animal's internal state (e.g., satiated for one of the two foods but not for the other) and so can differentially activate the representations of such two outcomes in NAccCo. Based on this, NAccCo can bias the selection of the currently valued goal within PFC.

The role of NAccCo in gPIT might be explained by the fact that its selections are non-specifically energised by a major efflux of dopamine to areas downstream of NAcc, e.g., DMS and the DLS, caused by the central nucleus of amygdala (CeA) via VTA (Cardinal et al., 2002b). In particular, gPIT experiments involve only one possible action at a time (vs. two of devaluation experiments). Thus, the presence of an additional conditioned stimulus recalling an additional appetitive outcome might energise the selection, and cause a more vigorous performance, of the action recalled by the selected goal (see Niv et al., 2007 for a review). So, in gPIT NAccCo contributes to select one specific goal but the DA produced by CeA via VTA might energise the selection and performance of the action associated with it.

Dopamine regulates the selection processes of NAccCo as in other portions of BG. Two distinct sub-populations of neurons can be distinguished in the striatum, one expressing low-affinity D1-like receptors and the other expressing high-affinity D2-like receptors (Gerfen et al., 1990; Floresco et al., 2003; Goto and Grace, 2005). D1-like and D2-like receptors are more concentrated in neurons within respectively the direct and indirect pathways of BG. Through a differential effect on the two types of receptors, and hence on the two pathways, higher levels of DA tend to produce an overall increase of the signal-to-noise ratio so sharpening the selection processes happening within the BG and hence NAccCo (see Gerfen, 2000 for a review and Gurney et al., 2001 for a computational model).

#### 3.5.6. Role of nucleus accumbens shell in goal selection

We now focus on NAccSh role in goal selection. According to our proposal, the NAccSh contributes to the selection of goals in a rather different but complementary way with respect to NAccCo, in particular by relying on its peculiar regulation of DA different from other regions of BG. Both NAccCo and NAccSh project directly and indirectly to midbrain DA systems—in particular to SNpc and VTA, respectively (see Figure 13). The direct projection is GABAergic and so tends to attenuate DA release. The indirect pathway, via VP, will, however, have a net excitatory effect (as VP is itself GABAergic; Floresco et al., 2003). In the circuit with NAccCo, the STN sends glutamatergic (excitatory) projections to VP which enhances its inhibitory effect on DA neurons in SNpc. STN also receives cortical input, thereby attenuating the dopaminergic response under cortical control. In contrast, the components of VP involved in the circuit with NAccSh do not receive STN input. This means that dopaminergic response in VTA, under control of NAccSh, is likely to be stronger than its counterpart in SNpc under control of NAccCo. We therefore hypothesise that a major role of NAccSh in processing value information that arrives there from Amg and Hip is to regulate dopaminergic tone based on this integrated value. VTA projects back to both NAccCo and NAccSh, and to areas of PFC which may be encoding goals, so NAccSh is in an ideal position to regulate the NAcc-PFC goal selection processes via DA. Interestingly, VTA dopaminergic projections also reach Amg (Cardinal et al., 2002a) and Hip (Lisman and Grace, 2005), and so also the activation and learning processes of these areas are influenced by NAccSh DA regulation.

We have seen above that the lack of the indirect pathway of NAccSh prevents it from implementing strong selections. Together with its important regulation of DA just reviewed, this implies that NAccSh contributes to goal selection in ways complementary to the BG-canonical selection of NAccCo. In this respect, we propose that NAccSh can contribute to augment the saliency of the goals selected by NAccCo, or to augment the saliency of multiple goals in parallel, with the support of DA regulation. Thus, experienced or predicted appetitive or novel stimuli might lead to enhanced salience of the selected goals, or to a multiple goal selection. The former might be useful to increase the vigor of the selection and performance of the actions that lead to pursuit of the selected goal (Salamone et al., 2003; Niv et al., 2007) given that NAccSh is at the vertex of the “dopaminergic spirals” involving striatum (Haber et al., 2000). The latter may instead be useful if multiple goals are hierarchically organised into distal goals and sub-goals that have to be selected at the same time, or when the performance of multiple goals is not in conflict (e.g., “eat and read” at the same time). Moreover, in situations involving novel and/or problem-solving conditions, a facilitated selection caused by a higher DA level may lead to an easier switching between goals in search of possible useful courses of actions (see Fiore et al., Submitted, for a model of this mechanism).

The ideas outlined above can explain the role of NAccSh in osPIT experiments. Thus, in a typical osPIT experiment NAccSh might receive information on the availability of one specific outcome, for example on the basis of the sight of the lever that produces it if pressed, and further activate the representation of such outcome on the basis of a conditioned stimulus previously associated with it through a Pavlovian training. In this case, the NAccSh DA control could support a *summation* of value from different sources related to the specific outcome resulting in the osPIT effects. This might also explain why lesions of NAccCo have no effect on osPIT: in contrast to NAccSh, NAccCo has the same canonical structure as the rest of BG, and so it can partially select a goal but not energise its selection beyond a certain level. It might also explain why lesions of NAccSh have no effect on devaluation: in this case, the standard BG selection performed by NAccCo is very effective, as one goal is value-charged while the other is devalued, while the capacity of NAccSh to over-activate specific selected outcomes is not relevant.

### 3.6. The prefrontal cortex: outcome representations

We now consider the fourth and last component of the system, the PFC, which supports the representation of different behavioral outcomes which the NAcc works on to select as the goal. Here we will mainly refer to rats, both in terms of anatomy and function, because more information on goal-directed behavior is available for this species. However, most considerations presented might be extended, at a general level, to primates, albeit with caution due to the differences between PFC in the two species (Preuss, 1995; Wise, 2008).

**Figure 14:**
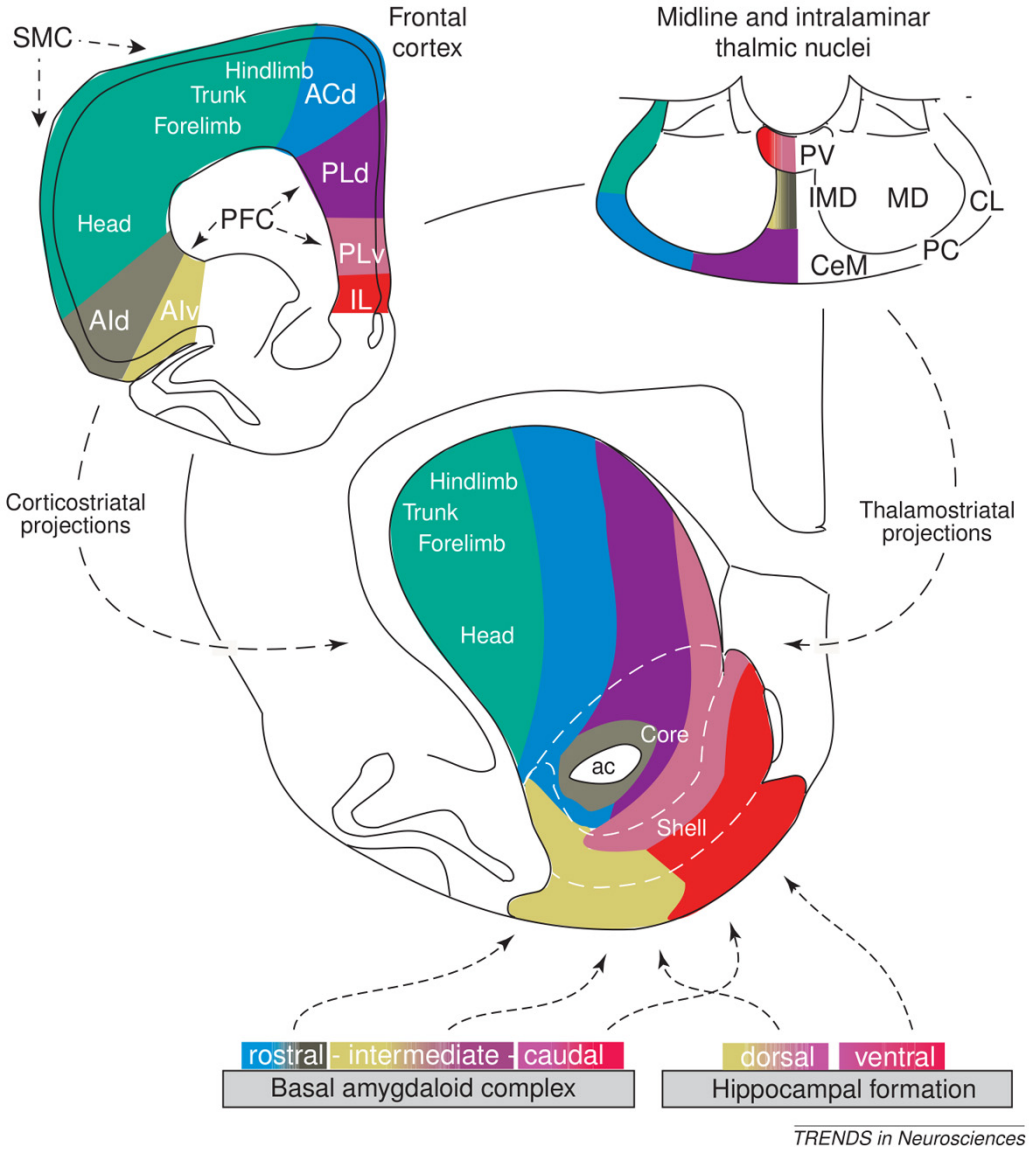
**Anatomy and connections of nucleus accumbens, basolateral amygdala, hippocampus, and prefrontal cortex**. Reprinted from Voorn et al. (2004), Copyright 2004, with permission from Elsevier.

NAcc forms loops with different PFC regions originating from three main sub-systems (Voorn et al., 2004; Humphries and Prescott, 2010; see also Figure 14). In particular, in rats NAcc forms loops with: (a) AIC; (b) PL; (c) IL (mainly with NAccSh). In primates, NAcc forms loops with (a) AIC *and* OFC; (b) PL (strongly connected with dlPFC); (c) IL *and* ACC (Haber et al., 1995; Chikama et al., 1997; Chiba et al., 2001). We make two proposals in regard to these anatomical observations. First, that each set of sub-systems (a–c) has a similar function in both species, and such function is empowered in primates. Second, that the three sub-systems have distinct but complementary roles in goal selection. These proposals are now articulated further.

System (a) in rats, comprising the NAcc-AIC loop, may implement goal selection on the basis of the mechanisms presented in section 3.5. In particular, the loop comprising NAccCo with AIC might have an important role in selecting distal or “ultimate,” biologically relevant goals such as the intake of food characterized by particular odors and tastes. The NAccSh-AIC loop might also contribute to the selection of ultimate goals by regulating their saliency on the basis of DA (section 3.5). Interestingly, primate OFC, which is adjacent to AIC, has a function in relation to odor and taste (Kringelbach and Rolls, 2004; Rolls, 2004a), and is also in loop with NAcc. We have seen in section 3.1 that OFC and BLA might form the augmented Pavlovian system of mammals capable of dealing with delayed outcomes (Schoenbaum et al., 1998; Rolls, 2004b). The acquisition of the NAcc-OFC loop in primates might have endowed them with the capacity to perform a more flexible selection of goals, based on the various sources of information received by NAcc, and the dynamic processes relying on the NAcc-OFC recurrent neural loops (Rolls, 2000b; Frank and Claus, 2006).

PL is a high-level area integrating various sources of information (from the hippocampal system, the PFC and sub-cortical areas) and on this basis it might form rich representations of stimuli (Sesack et al., 1989; Condé et al., 1995). The NAccCo-PL sub-system (b) might therefore support system (a) by selecting goals on the basis of their more abstract, auditory and visual features. Further, in primates, PL might have been augmented by the more complex dlPFC since, on the basis of its efferent projections, dlPFC seems to play functions analogous to, but more sophisticated than, those of PL (Vertes, 2004). In this regard, the dlPFC might encode more proximal sub-goals, i.e., goals in a “means-to-end” relation with the ultimate, more distal biologically-salient goals encoded primarily in PL and AIC (Miller and Cohen, 2001; Saito et al., 2005; Mushiake et al., 2006; see Solway and Botvinick, 2012, for a review).

Turning to sub-system (c), we propose that the role of the NAcc-IL system, and in particular of NAccSh-IL, is more subtle. Empirical evidence indicates that IL in rats is implicated in three different classes of behavioral effects. First, its projections to Amg (Quirk et al., 2003) are needed for the extinction of Pavlovian behaviors (Quirk and Mueller, 2008; Pape and Paré, 2010). Second, IL is also involved in the extinction of instrumental and Pavlovian behaviors (cf. Rhodes and Killcross, 2004; Peters et al., 2008). Third, IL is implicated in the switching from goal-directed to habitual behaviors due to overtraining, possibly via an inhibition of goal-directed behavior (as its lesion reinstates goal-directed strategies in overtrained rats Coutureau and Killcross, 2003). Overall, these data suggest that the NAcc-IL circuit might mainly contribute to inhibit Pavlovian, habitual, and goal-directed behaviors. In particular, the circuit might learn to inhibit Pavlovian reactions that are no longer useful, thanks to its inhibitory effects on Amg (Quirk and Mueller, 2008). With respect to instrumental behaviors, either habitual or goal-directed, when some behaviors are no longer useful (or even detrimental), NAcc might lead to no longer select them.

Interestingly, in primates the NAcc-ACC loop might enhance function (c): ACC is adjacent to IL and, in primates, it might play a different role from that in rats (where it mainly serves motor functions, see Cardinal et al., (2002a), similarly to posterior cingulate cortex of primates). Indeed, primate ACC has been shown to detect the missed achievement of expected desirable outcomes (Alexander and Brown, 2011). This detection might allow the NAcc-Acc loop to form inhibitory associations that prevent the selection of actions directed to unachievable/undesirable outcomes, so resulting in an empowerment of primates to inhibit behaviors in a goal-directed fashion.

Once goals have been selected by the NAcc-PFC system, they contribute to select the actions that accomplish them. Such actions are encoded in the systems formed by the sensorimotor cortical pathways (Cisek, 2007) and selected by the sensorimotor striato-cortical loops (Alexander et al., 1986; Mink, 1996). From a computational perspective, goal-based selection of actions performed by these systems is captured by the concept of *inverse model* (Gurney et al., 2012). An inverse model is a computational device that functionally links three elements: the *current state* of the environment and the animal, and the desired *future state* (the “goal”), with the *action* that might enable a transition from the current to the future state. The inverse model allows the recruitment of the action required to achieve the goal from the current state. According to the hypothesis proposed here, goals are mainly encoded in PFC and their value-based saliency is encoded in NAcc. In contrast, actions, intended as the sensorimotor transformations from proprioception to movements, are encoded in the neural pathways linking somatosensory cortex (SSC) to M1 (Pavlides et al., 1993; Tokimura et al., 2000). These actions are afforded by the current state of the environment (e.g., a lever might suggest “pressing” or “biting” action affordances to a rat). These affordances and the related motor plans are encoded in dorsal cortico-cortical neural streams (Goodale and Milner, 1992) linking associative sensory areas, in particular the PC, to motor areas, in particular the PMC (in turn linked to M1) (Jeannerod et al., 1995; Rizzolatti and Craighero, 2004; Cisek, 2007). The bridge from goal representations to action representations is supposedly based on links between PFC and PMC mediated by SMC (Nachev et al., 2008; Caligiore et al., 2013). The PFC is also strongly connected to PC which enables it to help action selection by selecting and modulating the representations of action affordances (Fogassi et al., 2005; Caligiore et al., 2010). In summary, cortical inverse models are formed by cortico-cortical connections (PFC-SMC-PMC-M1 and PFC-PC-PMC-M1) linking goal representations (PFC) to action-affordances and actions (PC-PMC-M1 and SSC-M1).

## 4. Discussion and conclusions

This paper proposed an hypothesis that explains how the brain selects goals on the basis of their current motivation value for the animal. Our hypothesis makes several key advances in our understanding of goal-directed behavior. First, it proposes a way for representing value from whatever source (amygdala, hippocampus) in a common currency, namely activity or salience within accumbens, thereby allowing all sources of value to be integrated and processed uniformly. This, in turn, allows us to hypothesise the idea that value, encoded in diverse structures like amygdala and hippocampus, can operate at the level of accumbens, to govern selection of goals in prefrontal cortex. Second, there are two principal mechanisms by which this process takes place: (a) biasing or gating specific goal representations in prefrontal cortex bidding for selection by the basal ganglia loop with accumbens core; (b) a somewhat diffuse facilitation of goals initiated at the level of accumbens shell and controlling release of dopamine in the shell and in cortex. Our scheme also emphasizes that goals encoded in prefrontal cortex recall actions based on action-outcome contingencies exploited as inverse models.

The pivotal component of the system, the ventral basal ganglia (nucleus accumbens), is the nexus between the value-charged representations in amygdala and hippocampus, and the representations of possible outcomes or goals in prefrontal cortex. The amygdala encodes stimuli having the valence of primary rewards and punishments, and hippocampus is capable of attributing a different type of valence to stimuli, namely its novelty. A further component of the system, developed later in evolution, is the prefrontal cortex. We proposed that, based on dynamical properties making them capable of storing information for seconds, key areas of prefrontal cortex initially evolved to enhance Pavlovian processes taking place in amygdala, and novelty processing in hippocampus. In a later evolutionary stage, prefrontal cortex became capable of encoding and selecting goals on the basis of a close dialog with accumbens.

Since the hypothesis specifies the function of the poorly understood limbic loop through basal ganglia, it also clarifies its relation to the “downstream” associative and sensorimotor loops. In particular, the accumbens, for which we have proposed a specific mechanistic account of its role in goal selection, is at the highest level of the striato-cortical loops hierarchy (Yin and Knowlton, 2006) and at the vertex of the “dopaminergic spirals” underlying motivational regulation (Haber et al., 2000). Through these means, accumbens plays a critical role in controlling and transmitting value information to the dorsomedial and dorsolateral compartments of the striatal hierarchy. This also explains why neural correlates of value are empirically found in ventral and DMS (see Balleine et al., 2008, for a review). Together with the idea that the areas of frontal cortex form whole computational systems with such basal ganglia districts, this also explains why several prefrontal cortex areas, most of which involved in goal selection by our proposal, have been found to activate in decision making tasks involving the accomplishment of valuable outcomes (see Knutson et al., 2009, for a review).

The hypothesis also reconciles several empirical findings on the different possible functions played by the accumbens (section 2.2). First, the hypothesis explains why the lesions of the amygdala and the accumbens core impair goal directed behavior (in particular, instrumental devaluation effects (Balleine et al., 2003). Indeed, the lesion of amygdala destroys the capacity to attribute value to goals, while the lesion of accumbens core eliminates the ability to select goals based on value. Second, it explains why the accumbens has been shown to be involved in motor functions (see Humphries and Prescott, 2010, for a review). In this respect, we have proposed that the primitive role of the accumbens was to support the performance of *innate* behaviors triggered by Pavlovian processes, especially those directed toward the outer world such as “approaching” and “orienting” behaviors (Cardinal et al., 2002b; Day et al., 2006; Gruber and McDonald, 2012). Building on this function, accumbens later acquired the capacity to also control *instrumentally acquired* behaviors via the selection of goals. Third, the hypothesis explains why accumbens plays a key role in “wanting” processes (Berridge, 2004) and energization of behavior (Salamone et al., 2003; Gruber and McDonald, 2012). In this respect, we have proposed that accumbens selects goals and attributes to them an incentive value regulated through its connections to the ventral tegmental area, in turn regulating the amount of dopamine injected in various key sub-cortical and cortical areas of brain (accumbens itself, amygdala, prefrontal cortex). Last, the hypothesis represents also a framework against which to explain the different functions that accumbens core and shell have in the transfer of Pavlovian motivational effects to instrumental behaviors (PIT). In particular, we have proposed that the execution of actions triggered on the basis of the selection of goals by accumbens core is affected by the aspecific amplifying effects of dopamine (aspecific Pavlovian to instrumental transfer effects, Corbit et al., 2007). In contrast, accumbens shell can cause focussed effects on the selection of goals (Corbit et al., 2007) thanks to its capacity to perform an unbounded summation of the incentive salience assigned to different goals from different sources (in particular, not only from the same goal but also from condition stimuli predicting them). Also these processes are affected by dopamine regulation.

Our hypothesis specifies in biological terms some aspects of the computational account of goal-directed behavior furnished by Daw et al., (2005). These authors propose that goal-directed behavior mechanisms can be captured by model-based reinforcement learning models. Our hypothesis specifies, in particular, one key aspect at the core of these models, namely the assignment of value to predicted states (outcomes), done by the amygdala/hippocampus-accumbens axis. Moreover (see section 3.6), the hypothesis can also be used to explain the role played in goal-directed behavior by internal models of the world (“transition function”), encoded in terms of action-outcome contingencies (cf. Mannella et al., 2010). Our proposal also furnishes a biologically detailed hypothesis on the brain systems that might correspond to the formal account of goal-directed behavior mechanisms presented by Solway and Botvinick (2012). These authors propose a Bayesian decomposition of the various processes involved in goal-directed behavior and link them to possible biological correspondents, but without supplying a view of how the whole integrated system might work (see Figure 6). In particular, the links between the biological components relied upon the relations between the elements of the model rather than on an analysis of their anatomical connections and functional dependencies based on biological evidence as done here (see section 3.1).

Finally, our scheme also reconciles various aspects of the other theoretical/computational proposals. In particular, it explains why accumbens core has been ascribed the role of actor and/or critic of model free models (e.g., Pennartz et al., 2011): this was based on its role in the expression of some motor behaviors, e.g., approaching and orienting (see explanation above), and on the correlation of its activity with the value assigned to ultimate goals, e.g., food receipt (here explained in terms of the information on value that amygdala and hippocampus send to accumbens). Our scheme also explains why the same interpretations attribute to the accumbens shell the role of critic of model free models: this is related to the role of shell in weighting the relative importance of different goals.

How can our theory on the role of hippocapus-accumbens connections in the assignment of novelty-based value to goals be reconciled with the proposal of their role in spatial navigation? There are two, possibly complementary, possibilities. According to the first, in a spatial context which is novel (e.g., a laboratory maze) hippocampus will initially respond to novelty. However, in normal laboratory situations even though the maze becomes familiar hippocampus will continue to respond because the outcomes encoded in hippocampus are valuable/rewarding, or connected to valuable/rewarding ultimate outcomes. A second possibility relies on the function of hippocampus as a temporary memory store. As seen above, hippocampus strongly responds to novel stimuli. This, and the related neuromodulatory events that follow, cause the Hip to encode those same stimuli. According to an important view on Hip function (McClelland et al., 1995; Rolls and Treves, 1998), however, hippocampus might work as a temporary store and the information it initially encodes might progressively transfer to cortex, possibly in an incomplete fashion. The response of hippocampus to no-longer-novel, but still not fully consolidated, spatial stimuli might so involve the period of transfer of stimuli to cortex. During this period, stimuli encoded in hippocampus might maintain a potential to be selected as goals so as to drive their further exploration (although with a lower priority).

The idea that novelty can be a source of motivation value, as we propose for hippocampus, is consistent with the novelty “exploration bonus” hypothesis, formalised in computational theories of reinforcement learning (Dayan and Sejnowski, 1994; Kakade and Dayan, 2002; Wittmann et al., 2007; Krebs et al., 2009). In this respect, Bunzeck et al., (2012) present evidence based on a brain-imaging study for which the Hippocampus and dopaminergic area plays a key role in modulating dopamine fostering exploration much like predicted by the exploration-bonus hypothesis [see also Niv et al., 2007]. With respect to this hypothesis, our proposal adds the notion of a *direct* role of accumbens in biasing goal selection within the prefrontal cortex based on the novelty of stimuli and outcomes or cues providing their anticipation.

Throughout this paper we have emphasised the notion of “decision making” about goals and actions as one which requires subcortical structures such as the basal ganglia. In contrast to this, much of the decision making literature emphasises the role of cortex (Shadlen and Newsome, 2001; Gold and Shadlen, 2007). These two views are not at odds with each other if we consider the relation between *perceptual* decision making and its translation into *action* for response (whether it is in a laboratory task or a real-world situation). The two views have been brought together in recent work on decision making and basal ganglia (Bogacz and Gurney, 2007; Lepora and Gurney, 2012). Essentially, the idea is that evidence for a decision about a particular percept is accumulated as cortical activity in high level sensory areas and that this is used as input into basal ganglia working as a selection engine for action. Indeed, in some interpretations, the accumulation itself is mediate by basal ganglia (Bogacz and Larsen, 2011). In this view, the salience of a goal or action might be interpreted as evidence for a decision to enact that goal or action.

The scheme presented here is able to make empirically testable predictions. One key claim of the model is that the value attributed to goals is generated by amygdala and hippocampus, and this value is integrated in NAcc in a “common currency.” The instrumental devaluation experiments already support the generation of appetitive/aversive value by amygdala. It would, however, be possible to manipulate *both* appetitive and novelty aspects of outcomes in instrumental experiments: our theory would predict that both novelty and appetitive aspects of outcomes are relevant for their selection, and also that there is a trade-off between the two. Thus, for example, it should be possible to lesion the accumbens and see if (as we predict) it is important for selecting novel vs. familiar (neutral) outcomes as it is for selecting appetitive outcomes with a higher-value in devaluation experiments. Further experiments might use two goals with the same appetitive value and different novelty value, and test the behavioral attractiveness of the two goals.

Other predictions relate to the differential role of accumbens core and shell in selecting goals. For example, accumbens core goal selection processes should be less sensitive to dopamine depletion than those of accumbens shell. This could be tested with Pavlovian-instrumental transfer experiments.

Finally we make predictions about the function of the NAcc-prefrontal cortex loops to select goals. It would be possible, for example, to run instrumental experiments after lesioning prefrontal cortex areas such as agranular insular cortex (and/or orbitofrontal cortex in primates), or prelimbic cortex (and/or dorsolateral prefrontal cortex in primates), or infralimbic cortex (and/or anterior cingulate cortex in primates) and test the animals with different outcomes that vary in terms of their sensorial features (e.g., taste/odor vs. visual/auditive features) which we have proposed are processed separately in those different areas.

Notwithstanding the explanatory and predictive power of our hypothesis, various issues related to it deserve further investigations in future work. We highlight these issues in the form of a list of questions:

- What is the dynamical interplay between the novelty and appetitive/aversive value of stimuli during learning, and in particular during their passage from novel to familiar?
- What is the specific interplay between the selection processes of accumbens core and shell, and their interdependencies based on the direct connections existing between them?
- Is there a quantitative relation between dopamine levels in accumbens and the importance of goals? What are the specific mechanisms that support the regulation of such levels based on the loops existing between accumbens and dopaminergic areas?
- How are representations of outcomes formed in prefrontal cortex and in accumbens, and how do they get connected between them and with the representations in amygdala and hippocampus?
- What are the specific mechanisms that lead to the formation of action-outcome cortical/sub-cortical inverse/forward models?

## Conflict of interest statement

The authors declare that the research was conducted in the absence of any commercial or financial relationships that could be construed as a potential conflict of interest.

## Appendix

**Table A1:** **Acronyms used in the paper to indicate various components of brain**.

ACC	Anterior cingulate cortex
AIC	Agranular insular cortex
Amg	Amygdala
BG	Basal ganglia
BLA	Amygdala, basolateral complex
CA1	Hippocampus, cornu Ammonis region 1
CA3	Hippocampus, cornu Ammonis region 3
CeA	Amygdala, central nucleus
DG	Hippocampus, dental girus
dlPFC	Dorsolateral prefrontal cortex
DLS	Dorsolateral striatum
DMS	Dorsomedial striatum
EC	Entorhinal cortex
FEF	Frontal eye fields
GPe	Globus pallidus, external division
GPi	Globus pallidus, internal division
Hip	Hippocampus
IL	Infralimbic cortex
ITC	Inferotemporal cortex
M1	Primary motor cortex
NAcc	Nucleus accumbens
NAccCo	Nucleus accumbens, core part
NAccSh	Nucleus accumbens, shell part
OFC	Orbitofrontal cortex
PFC	Prefrontal cortex
PL	Prelimbic cortex
PC	Parietal cortex
PMC	Premotor cortex
SMC	Supplementary motor cortex
SNpc	Substantia nigra, pars compacta
SNpr	substantia nigra, pars reticulata
SSC	Somatosensory cortex
STN	Subthalamic nucleus
Sub	Hippocamus, subiculum
VTA	Ventral tegmental area

## References

[B1] Alexander G. E. DeLong M. R. Strick P. L. (1986). Parallel organization of functionally segregated circuits linking basal ganglia and cortex. Annu. Rev. Neurosci. 9, 357–381 10.1146/annurev.ne.09.030186.002041 3085570

[B2] Alexander W. H. Brown J. W. (2011). Medial prefrontal cortex as an action-outcome predictor (supplementary material). Nat. Neurosci. 14, e1–e6 10.1038/nn.2921 21926982 PMC3183374

[B3] Baldassarre G. Mannella F. Fiore V. G. Redgrave P. Gurney K. Mirolli M. (2012). Intrinsically motivated action-outcome learning and goal-based action recall: a system-level bio-constrained computational model. Neural Netw. 41, 168–187 10.1016/j.neunet.2012.09.015 23098753

[B4] Baldassarre G. (2002). Planning with Neural Networks and Reinforcement Learning. Ph.D. thesis, Department of Computer Science, University of Essex.

[B5] Balleine B. W. Daw N. D. O'Doherty J. P. (2008). Multiple forms of value learning and the function of dopamine, in Neuroeconomics: Decision Making and the Brain, eds Glimcher P. W. Camerer C. F. Fehr E. Poldrack R. A. (London: Academic Press), 367–385

[B6] Balleine B. W. Delgado M. R. Hikosaka O. (2007). The role of the dorsal striatum in reward and decision-making. J. Neurosci. 27, 8161–8165 10.1523/JNEUROSCI.1554-07.2007 17670959 PMC6673072

[B7] Balleine B. W. Dickinson A. (1998). Goal-directed instrumental action: contingency and incentive learning and their cortical substrates. Neuropharmacology 37, 407–419 10.1016/S0028-3908(98)00033-1 9704982

[B8] Balleine B. W. Killcross S. (2006). Parallel incentive processing: an integrated view of amygdala function. Trends Neurosci. 29, 272–279 10.1016/j.tins.2006.03.002 16545468

[B9] Balleine B. W. Killcross S. A. Dickinson A. (2003). The effect of lesions of the basolateral amygdala on instrumental conditioning. J. Neurosci. 23, 666–675 12533626 10.1523/JNEUROSCI.23-02-00666.2003 PMC6741878

[B10] Balleine B. W. Liljeholm M. Ostlund S. B. (2009). The integrative function of the basal ganglia in instrumental conditioning. Behav. Brain Res. 199, 43–52 10.1016/j.bbr.2008.10.034 19027797

[B11] Balleine B. W. Ostlund S. B. (2007). Still at the choice-point: action selection and initiation in instrumental conditioning. Ann. N.Y. Acad. Sci. 1104, 147–171 10.1196/annals.1390.006 17360797

[B12] Bandler R. Keay K. A. Floyd N. Price J. (2000). Central circuits mediating patterned autonomic activity during active vs. passive emotional coping. Brain Res. Bull. 53, 95–104 10.1016/S0361-9230(00)00313-0 11033213

[B13] Bast T. (2007). Toward an integrative perspective on hippocampal function: from the rapid encoding of experience to adaptive behavior. Rev. Neurosci. 18, 253–281 18019609 10.1515/revneuro.2007.18.3-4.253

[B14] Behbehani M. M. (1995). Functional characteristics of the midbrain periaqueductal gray. Prog. Neurobiol. 46, 575–605 10.1016/0301-0082(95)00009-K 8545545

[B15] Berridge K. C. (2004). Motivation concepts in behavioral neuroscience. Physiol. Behav. 81, 179–209 10.1016/j.physbeh.2004.02.004 15159167

[B16] Berridge K. C. (2007). The debate over dopamine's role in reward: the case for incentive salience. Psychopharmacology 191, 391–431 10.1007/s00213-006-0578-x 17072591

[B17] Bertsekas D. P. (1987). Dynamic Programming: Deterministic and Stochastic Models. Englewood Cliffs, NJ: Prentice Hall

[B18] Bird C. M. Burgess N. (2008). The hippocampus and memory: insights from spatial processing. Nat. Rev. Neurosci. 9, 182–194 10.1038/nrn2335 18270514

[B19] Bogacz R. Gurney K. (2007). The basal ganglia and cortex implement optimal decision making between alternative actions. Neural Comput. 19, 442–477 10.1162/neco.2007.19.2.442 17206871

[B20] Bogacz R. Larsen T. (2011). Integration of reinforcement learning and optimal decision-making theories of the basal ganglia. Neural Comput. 23, 817–851 10.1162/NECO_a_00103 21222528

[B21] Bornstein A. Daw N. (2011). Multiplicity of control in the basal ganglia: computational roles of striatal subregions. Curr. Opin. Neurobiol. 21, 374–380 10.1016/j.conb.2011.02.009 21429734 PMC3269306

[B22] Botvinick M. M. Niv Y. Barto A. C. (2008). Hierarchically organized behavior and its neural foundations: a reinforcement learning perspective. Cognition. 113, 262–280 10.1016/j.cognition.2008.08.011 18926527 PMC2783353

[B23] Bunzeck N. Doeller C. F. Dolan R. J. Duzel E. (2012). Contextual interaction between novelty and reward processing within the mesolimbic system. Hum. Brain Mapp. 33, 1309–1324 10.1002/hbm.21288 21520353 PMC3498733

[B24] Burnod Y. Baraduc P. Battaglia-Mayer A. Guigon E. Koechlin E. Ferraina S. (1999). Parieto-frontal coding of reaching: an integrated framework. Exp. Brain Res. 129, 325–346 10.1007/s002210050902 10591906

[B25] Cabib S. Puglisi-Allegra S. (2012). The mesoaccumbens dopamine in coping with stress. Neurosci. Biobehav. Rev. 36, 79–89 10.1016/j.neubiorev.2011.04.012 21565217

[B26] Caligiore D. Borghi A. Parisi D. Baldassarre G. (2010). Tropicals: a computational embodied neuroscience model of compatibility effects. Psycol. Rev. 117, 1188–1228 10.1037/a0020887 21038976

[B27] Caligiore D. Borghi A. Parisi D. Ellis R. Cangelosi A. Baldassarre G. (2013). How affordances associated with a distractor object affect compatibility effects: a study with the computational model tropicals. Psychol. Res. 77, 7–19 10.1007/s00426-012-0424-1 22327121

[B28] Cardinal R. N. Parkinson J. A. Hall J. Everitt B. J. (2002a). Emotion and motivation: the role of the amygdala, ventral striatum, and prefrontal cortex. Neurosci. Biobehav. Rev. 26, 321–352 12034134 10.1016/s0149-7634(02)00007-6

[B29] Cardinal R. N. Parkinson J. A. Lachenal G. Halkerston K. M. Rudarakanchana N. Hall J. (2002b). Effects of selective excitotoxic lesions of the nucleus accumbens core, anterior cingulate cortex, and central nucleus of the amygdala on autoshaping performance in rats. Behav. Neurosci. 116, 553–567 12148923 10.1037//0735-7044.116.4.553

[B30] Cavigelli S. A. McClintock M. K. (2003). Fear of novelty in infant rats predicts adult corticosterone dynamics and an early death. Proc. Natl. Acad. Sci. U.S.A. 100, 16131–16136 10.1073/pnas.2535721100 14673078 PMC307704

[B31] Cheatwood J. L. Reep R. L. Corwin J. V. (2003). The associative striatum: cortical and thalamic projections to the dorsocentral striatum in rats. Brain Res. 968, 1–14 10.1016/S0006-8993(02)04212-9 12644259

[B32] Chevalier G. Deniau J. M. (1990). Disinhibition as a basic process in the expression of striatal functions. Trends Neurosci. 13, 277–280 10.1016/0166-2236(90)90109-N 1695403

[B33] Chiba T. Kayahara T. Nakano K. (2001). Efferent projections of infralimbic and prelimbic areas of the medial prefrontal cortex in the japanese monkey, *macaca fuscata*. Brain Res. 888, 83–101 10.1016/S0006-8993(00)03013-4 11146055

[B34] Chikama M. McFarland N. R. Amaral D. G. Haber S. N. (1997). Insular cortical projections to functional regions of the striatum correlate with cortical cytoarchitectonic organization in the primate. J. Neurosci. 17, 9686–9705 9391023 10.1523/JNEUROSCI.17-24-09686.1997 PMC6573402

[B35] Cisek P. (2007). Cortical mechanisms of action selection: the affordance competition hypothesis. Philos. Trans. R. Soc. Lond. B. Biol. Sci. 362, 1585–1599 10.1098/rstb.2007.2054 17428779 PMC2440773

[B36] Condé F. Maire-Lepoivre E. Audinat E. Crépel F. (1995). Afferent connections of the medial frontal cortex of the rat. ii. Cortical and subcortical afferents. J. Compt. Neurol. 352, 567–593 10.1002/cne.903520407 7722001

[B37] Corbit L. H. Balleine B. W. (2003). The role of prelimbic cortex in instrumental conditioning. Behav. Brain Res. 146, 145–157 10.1016/j.bbr.2003.09.023 14643467

[B38] Corbit L. H. Balleine B. W. (2005). Double dissociation of basolateral and central amygdala lesions on the general and outcome-specific forms of pavlovian-instrumental transfer. J. Neurosci. 25, 962–970 10.1523/JNEUROSCI.4507-04.2005 15673677 PMC6725628

[B39] Corbit L. H. Balleine B. W. (2011). The general and outcome-specific forms of pavlovian-instrumental transfer are differentially mediated by the nucleus accumbens core and shell. J. Neurosci. 31, 11786–11794 10.1523/JNEUROSCI.2711-11.2011 21849539 PMC3208020

[B40] Corbit L. H. Janak P. H. Balleine B. W. (2007). General and outcome-specific forms of pavlovian-instrumental transfer: the effect of shifts in motivational state and inactivation of the ventral tegmental area. Eur. J. Neurosci. 26, 3141–3149 10.1111/j.1460-9568.2007.05934.x 18005062

[B41] Corbit L. H. Muir J. L. Balleine B. W. (2001). The role of the nucleus accumbens in instrumental conditioning: evidence of a functional dissociation between accumbens core and shell. J. Neurosci. 21, 3251–3260 11312310 10.1523/JNEUROSCI.21-09-03251.2001 PMC6762583

[B42] Corbit L. H. Muir J. L. Balleine B. W. (2003). Lesions of mediodorsal thalamus and anterior thalamic nuclei produce dissociable effects on instrumental conditioning in rats. Eur. J. Neurosci. 18, 1286–1294 10.1046/j.1460-9568.2003.02833.x 12956727

[B43] Coutureau E. Killcross S. (2003). Inactivation of the infralimbic prefrontal cortex reinstates goal-directed responding in overtrained rats. Behav. Brain Res. 146, 167–174 10.1016/j.bbr.2003.09.025 14643469

[B44] Dalley J. W. Cardinal R. N. Robbins T. W. (2004). Prefrontal executive and cognitive functions in rodents: neural and neurochemical substrates. Neurosci. Biobehav. Rev. 28, 771–784 10.1016/j.neubiorev.2004.09.006 15555683

[B45] Davidson R. E. Richardson A. M. (1970). Classical conditioning of skeletal and autonomic responses in the lizard (*crotaphytus collaris*). Physiol. Behav. 5, 589–594 10.1016/0031-9384(70)90085-5 5535515

[B46] Davis M. Whalen P. J. (2001). The amygdala: vigilance and emotion. Mol. Psychiatry 6, 13–34 10.1038/sj.mp.4000812 11244481

[B47] Daw N. D. Niv Y. Dayan P. (2005). Uncertainty-based competition between prefrontal and dorsolateral striatal systems for behavioral control. Nat. Neurosci. 8, 1704–1711 10.1038/nn1560 16286932

[B48] Day J. J. Carelli R. M. (2007). The nucleus accumbens and Pavlovian reward learning. Neuroscientist 13, 148–159 10.1177/1073858406295854 17404375 PMC3130622

[B49] Day J. J. Wheeler R. A. Roitman M. F. Carelli R. M. (2006). Nucleus accumbens neurons encode Pavlovian approach behaviors: evidence from an autoshaping paradigm. Eur. J. Neurosci. 23, 1341–1351 10.1111/j.1460-9568.2006.04654.x 16553795

[B163] Dayan P. Sejnowski T. J. (1994). Td(λ) converges with probability 1. Mach. Learn. 14, 295–301

[B140] Dickinson A. Balleine B. (1994). Motivational control of goal-directed action. Anim. Learn. Behav. 22, 1–18 10.3758/BF03199951

[B101] Eichenbaum H. Dudchenko P. Wood E. Shapiro M. Tanila H. (1999). The hippocampus, memory, and place cells: is it spatial memory or a memory space? Neuron 23, 209–226 10399928 10.1016/s0896-6273(00)80773-4

[B91] Euston D. R. Gruber A. J. McNaughton B. L. (2012). The role of medial prefrontal cortex in memory and decision making. Neuron 76, 1057–1070 10.1016/j.neuron.2012.12.002 23259943 PMC3562704

[B61] Faure A. Haberland U. Condé F. Massioui N. E. (2005). Lesion to the nigrostriatal dopamine system disrupts stimulus-response habit formation. J. Neurosci. 25, 2771–2780 10.1523/JNEUROSCI.3894-04.2005 15772337 PMC6725127

[B57] Featherstone R. E. McDonald R. J. (2004). Dorsal striatum and stimulus-response learning: lesions of the dorsolateral, but not dorsomedial, striatum impair acquisition of a stimulus-response-based instrumental discrimination task, while sparing conditioned place preference learning. Neuroscience 124, 23–31 10.1016/j.neuroscience.2003.10.038 14960336

[B56] Finch D. M. (1996). Neurophysiology of converging synaptic inputs from the rat prefrontal cortex, amygdala, midline thalamus, and hippocampal formation onto single neurons of the caudate/putamen and nucleus accumbens. Hippocampus 6, 495–512 10.1002/(SICI)1098-1063(1996)6:5<495::AID-HIPO3>3.3.CO;2-I 8953303

[B58] Floresco S. B. (2007). Dopaminergic regulation of limbic-striatal interplay. J. Psychiatry Neurosci. 32, 400–411 18043763 PMC2077353

[B59] Floresco S. B. West A. R. Ash B. Moore H. Grace A. A. (2003). Afferent modulation of dopamine neuron firing differentially regulates tonic and phasic dopamine transmission. Nat. Neurosci. 6, 968–973 10.1038/nn1103 12897785

[B60] Fogassi L. Ferrari P. F. Gesierich B. Rozzi S. Chersi F. Rizzolatti G. (2005). Parietal lobe: from action organization to intention understanding. Science 308, 662–667 10.1126/science.1106138 15860620

[B61a] Frank M. J. Claus E. D. (2006). Anatomy of a decision: striato-orbitofrontal interactions in reinforcement learning, decision making, and reversal. Psychol. Rev. 113, 300–326 10.1037/0033-295X.113.2.300 16637763

[B62] Frankland P. W. Bontempi B. (2005). The organization of recent and remote memories. Nat. Rev. Neurosci. 6, 119–130 10.1038/nrn1607 15685217

[B63] Frey U. Morris R. G. (1998). Weak before strong: dissociating synaptic tagging and plasticity-factor accounts of late-ltp. Neuropharmacology 37, 545–552 10.1016/S0028-3908(98)00040-9 9704995

[B64] Fudge J. L. Emiliano A. B. (2003). The extended amygdala and the dopamine system: another piece of the dopamine puzzle. J. Neuropsychiatry Clin. Neurosci. 15, 306–316 10.1176/appi.neuropsych.15.3.306 12928506 PMC2394680

[B65] Fudge J. L. Haber S. N. (2000). The central nucleus of the amygdala projection to dopamine subpopulations in primates. Neuroscience 97, 479–494 10.1016/S0306-4522(00)00092-0 10828531

[B66] Funahashi S. (2001). Neuronal mechanisms of executive control by the prefrontal cortex. Neurosci. Res. 39, 147–165 10.1016/S0168-0102(00)00224-8 11223461

[B67] Fuster J. M. (1997). The Prefrontal Cortex: Anatomy, Physiology, and Neuropsychology of the Frontal Lobe. 3rd Edn Philadelphia, PA: Lippincott-Raven

[B68] Gasbarri A. Sulli A. Packard M. G. (1997). The dopaminergic mesencephalic projections to the hippocampal formation in the rat. Prog. Neuropsychopharmacol. Biol. Psychiatry 21, 1–22 10.1016/S0278-5846(96)00157-1 9075256

[B69] Gerfen C. R. (2000). Molecular effects of dopamine on striatal-projection pathways. Trends Neurosci. 23(10 Suppl.), S64–S70 10.1016/S1471-1931(00)00019-7 11052222

[B70] Gerfen C. R. Engber T. M. Mahan L. C. Susel Z. Chase T. N. Monsma F. J. (1990). D1 and d2 dopamine receptor-regulated gene expression of striatonigral and striatopallidal neurons. Science 250, 1429–1432 10.1126/science.2147780 2147780

[B71] Glimcher P. W. Camerer C. F. Fehr E. Poldrack R. A. (2010). Neuroeconomics: Decision Making and the Brain. Amsterdam: Academic Press

[B72] Gold J. I. Shadlen M. N. (2007). The neural basis of decision making. Annu. Rev. Neurosci. 30, 535–574 10.1146/annurev.neuro.29.051605.113038 17600525

[B73] Goodale M. A. Milner A. D. (1992). Separate visual pathways for perception and action. Trends Neurosci. 15, 20–25 10.1016/0166-2236(92)90344-8 1374953

[B74] Goto Y. Grace A. A. (2005). Dopaminergic modulation of limbic and cortical drive of nucleus accumbens in goal-directed behavior. Nat. Neurosci. 8, 805–812 10.1038/nn1471 15908948

[B75] Goto Y. O'Donnell P. (2002). Timing-dependent limbic-motor synaptic integration in the nucleus accumbens. Proc. Natl. Acad. Sci. U.S.A. 99, 13189–13193 10.1073/pnas.202303199 12237410 PMC130608

[B76] Gruber A. J. McDonald R. J. (2012). Context, emotion, and the strategic pursuit of goals: interactions among multiple brain systems controlling motivated behavior. Front. Behav. Neurosci. 6:50 10.3389/fnbeh.2012.00050 22876225 PMC3411069

[B194] Gurney K. Lepora N. Shah A. Koene A. Redgrave P. (2012). Action discovery and intrinsic motivation: a biologically constrained formalisation, in Intrinsically Motivated Learning in Natural and Artificial Systems, eds Baldassarre G. Mirolli M. Baldassarre G. Mirolli M. (Berlin: Springer-Verlag).

[B186] Gurney K. Prescott T. Redgrave P. (2001). A computational model of action selection in the basal ganglia. i. A new functional anatomy. Biol. Cybern. 84, 401–410 10.1007/PL00007984 11417052

[B79] Haber S. N. Fudge J. L. McFarland N. R. (2000). Striatonigrostriatal pathways in primates form an ascending spiral from the shell to the dorsolateral striatum. J. Neurosci. 20, 2369–2382 10704511 10.1523/JNEUROSCI.20-06-02369.2000 PMC6772499

[B80] Haber S. N. Kunishio K. Mizobuchi M. Lynd-Balta E. (1995). The orbital and medial prefrontal circuit through the primate basal ganglia. J. Neurosci. 15(7 Pt 1), 4851–4867 7623116 10.1523/JNEUROSCI.15-07-04851.1995 PMC6577885

[B81] Hall J. Parkinson J. A. Connor T. M. Dickinson A. Everitt B. J. (2001). Involvement of the central nucleus of the amygdala and nucleus accumbens core in mediating pavlovian influences on instrumental behavior. Eur. J. Neurosci. 13, 1984–1992 10.1046/j.0953-816x.2001.01577.x 11403692

[B82] Hasselmo M. E. Schnell E. Barkai E. (1995). Dynamics of learning and recall at excitatory recurrent synapses and cholinergic modulation in rat hippocampal region ca3. J. Neurosci. 15(7 Pt 2), 5249–5262 7623149 10.1523/JNEUROSCI.15-07-05249.1995 PMC6577857

[B83] Hatfield T. Han J. S. Conley M. Gallagher M. Holland P. (1996). Neurotoxic lesions of basolateral, but not central, amygdala interfere with pavlovian second-order conditioning and reinforcer devaluation effects. J. Neurosci. 16, 5256–5265 8756453 10.1523/JNEUROSCI.16-16-05256.1996 PMC6579315

[B84] Hikosaka O. Takikawa Y. Kawagoe R. (2000). Role of the basal ganglia in the control of purposive saccadic eye movements. Physiol. Rev. 80, 953–978 10893428 10.1152/physrev.2000.80.3.953

[B85] Hok V. Save E. Lenck-Santini P. P. Poucet B. (2005). Coding for spatial goals in the prelimbic/infralimbic area of the rat frontal cortex. Proc. Natl. Acad. Sci. U.S.A. 102, 4602–4607 10.1073/pnas.0407332102 15761059 PMC555486

[B86] Horvitz J. C. (2000). Mesolimbocortical and nigrostriatal dopamine responses to salient non-reward events. Neuroscience 96, 651–656 10.1016/S0306-4522(00)00019-1 10727783

[B87] Houk J. C. Adams J. L. Barto A. G. (1995a). A model of how the basal ganglia generate and use neural signals that predict reinforcement, in Models of Information Processing in the Basal Ganglia, eds Houk J. C. Davis J. L. Beiser D. G. (Cambridge, MA: MIT Press), 249–270

[B88] Houk J. C. Davids J. L. Beiser D. G. (1995b). Models of Information Processing in the Basal Ganglia. Cambridge, MA: The MIT Press

[B89] Humphries M. D. Gurney K. N. (2002). The role of intra-thalamic and thalamocortical circuits in action selection. Network 13, 131–156 10.1080/net.13.1.131.156 11873842

[B90] Humphries M. D. Prescott T. J. (2010). The ventral basal ganglia, a selection mechanism at the crossroads of space, strategy, and reward. Prog. Neurobiol. 90, 385–417 10.1016/j.pneurobio.2009.11.003 19941931

[B91a] Izquierdo A. Suda R. K. Murray E. A. (2004). Bilateral orbital prefrontal cortex lesions in rhesus monkeys disrupt choices guided by both reward value and reward contingency. J. Neurosci. 24, 7540–7548 10.1523/JNEUROSCI.1921-04.2004 15329401 PMC6729636

[B92] Jeannerod M. Arbib M. A. Rizzolatti G. Sakata H. (1995). Grasping objects: the cortical mechanisms of visuomotor transformation. Trends Neurosci. 18, 314–320 10.1016/0166-2236(95)93921-J 7571012

[B93] Joel D. Niv Y. Ruppin E. (2002). Actor-critic models of the basal ganglia: new anatomical and computational perspectives. Neural Netwo. 15, 535–547 10.1016/S0893-6080(02)00047-3 12371510

[B94] Johnson A. Redish A. D. (2007). Neural ensembles in ca3 transiently encode paths forward of the animal at a decision point. J. Neurosci. 27, 12176–12189 10.1523/JNEUROSCI.3761-07.2007 17989284 PMC6673267

[B95] Johnson A. van der Meer M. A. A. Redish A. D. (2007). Integrating hippocampus and striatum in decision-making. Curr. Opin. Neurobiol. 17, 692–697 10.1016/j.conb.2008.01.003 18313289 PMC3774291

[B96] Kakade S. Dayan P. (2002). Dopamine: generalization and bonuses. Neural Netw. 15, 549–559 10.1016/S0893-6080(02)00048-5 12371511

[B97] Karlsson M. P. Frank L. M. (2008). Network dynamics underlying the formation of sparse, informative representations in the hippocampus. J. Neurosci. 28, 14271–14281 10.1523/JNEUROSCI.4261-08.2008 19109508 PMC2632980

[B98] Khamassi M. Humphries M. (2012). Integrating cortico-limbic-basal ganglia architectures for learning model-based and model-free navigation strategies. Front. Behav. Neurosci. 6:79 10.3389/fnbeh.2012.00079 23205006 PMC3506961

[B99] Knutson B. Delgado M. R. Phillips P. E. (2009). Representation of subjective value in the striatum, in Neuroeconomics – Decision Making and the Brain, eds Glimcher P. W. Camerer C. F. Fehr E. Poldrack R. A. (Amsterdam: Elsevier), 389–406

[B100] Krebs R. M. Schott B. H. Schütze H. Düzel E. (2009). The novelty exploration bonus and its attentional modulation. Neuropsychologia 47, 2272–2281 10.1016/j.neuropsychologia.2009.01.015 19524091

[B101a] Kringelbach M. L. Rolls E. T. (2004). The functional neuroanatomy of the human orbitofrontal cortex: evidence from neuroimaging and neuropsychology. Prog. Neurobiol. 72, 341–372 10.1016/j.pneurobio.2004.03.006 15157726

[B102] Kumaran D. Maguire E. A. (2005). The human hippocampus: cognitive maps or relational memory? J. Neurosci. 25, 7254–7259 10.1523/JNEUROSCI.1103-05.2005 16079407 PMC6725222

[B103] Kumaran D. Maguire E. A. (2007). Match mismatch processes underlie human hippocampal responses to associative novelty. J. Neurosci. 27, 8517–8524 10.1523/JNEUROSCI.1677-07.2007 17687029 PMC2572808

[B104] Lepora N. F. Gurney K. N. (2012). The basal ganglia optimize decision making over general perceptual hypotheses. Neural. Comput. 24, 2924–2945 10.1162/NECO_a_00360 22920846

[B105] Lewis J. Chambers J. M. Redgrave P. Gurney K. N. (2011). A computational model of interconnected basal ganglia-thalamocortical loops for goal directed action sequences. BMC Neurosci. 12(Suppl. 1):136 10.1186/1471-2202-12-S1-P136

[B106] Lex B. Hauber W. (2010). The role of nucleus accumbens dopamine in outcome encoding in instrumental and pavlovian conditioning. Neurobiol. Learn Mem. 93, 283–290 10.1016/j.nlm.2009.11.002 19931626

[B107] Lisman J. Grace A. A. Duzel E. (2011). A neohebbian framework for episodic memory; role of dopamine-dependent late LTP. Trends Neurosci. 34, 536–547 10.1016/j.tins.2011.07.006 21851992 PMC3183413

[B108] Lisman J. E. Grace A. A. (2005). The hippocampal-vta loop: controlling the entry of information into long-term memory. Neuron 46, 703–713 10.1016/j.neuron.2005.05.002 15924857

[B109] Lisman J. E. Otmakhova N. A. (2001). Storage, recall, and novelty detection of sequences by the hippocampus: elaborating on the socratic model to account for normal and aberrant effects of dopamine. Hippocampus 11, 551–568 10.1002/hipo.1071 11732708

[B110] Lubow R. E. Moore A. U. (1959). Latent inhibition: the effect of nonreinforced pre-exposure to the conditional stimulus. J. Comp. Physiol. Psychol. 52, 415–419 10.1037/h0046700 14418647

[B111] Lucas G. A. Deich J. D. Wasserman E. A. (1981). Trace autoshaping: acquisition, maintenance, and path dependence at long trace intervals. J. Exp. Anal. Behav. 36, 61–74 10.1901/jeab.1981.36-61 16812232 PMC1333053

[B112] Mannella F. Koene A. Baldassarre G. (2009). Navigation via pavlovian conditioning: a robotic bio-constrained model of autoshaping in rats, in Proceedings of the Ninth International Conference on Epigenetic Robotics (EpiRob2009). Vol. 146 12–14 November 2009 (Lund University, Lund), 97–104

[B113] Mannella F. Mirolli M. Baldassarre G. (2010). The interplay of pavlovian and instrumental processes in devaluation experiments: a computational embodied neuroscience model tested with a simulated rat, in Modelling Perception With Artificial Neural Networks, eds Tosh C. Ruxton G. (Cambridge: Cambridge University Press), 93–113 10.1017/CBO9780511779145.006

[B114] Matsumoto K. Tanaka K. (2004). The role of the medial prefrontal cortex in achieving goals. Curr. Opin. Neurobiol. 14, 178–185 10.1016/j.conb.2004.03.005 15082322

[B115] McClelland J. L. McNaughton B. L. O'Reilly R. C. (1995). Why there are complementary learning systems in the hippocampus and neocortex: insights from the successes and failures of connectionist models of learning and memory. Psychol. Rev. 102, 419–457 10.1037/0033-295X.102.3.419 7624455

[B116] McClure S. M. Daw N. D. Montague P. R. (2003). A computational substrate for incentive salience. Trends Neurosci. 26, 423–428 10.1016/S0166-2236(03)00177-2 12900173

[B117] McDonald R. J. White N. M. (1993). A triple dissociation of memory systems: hippocampus, amygdala, and dorsal striatum. Behav. Neurosci. 107, 3–22 10.1037/0735-7044.107.1.3 8447956

[B118] Medina J. F. Repa C. J. Mauk M. D. LeDoux J. E. (2002). Parallels between cerebellum- and amygdala-dependent conditioning. Nat. Rev. Neurosci. 3, 122–131 10.1038/nrn728 11836520

[B119] Meeter M. Murre J. M. J. Talamini L. M. (2004). Mode shifting between storage and recall based on novelty detection in oscillating hippocampal circuits. Hippocampus 14, 722–741 10.1002/hipo.10214 15318331

[B120] Meyer F. F. Louilot A. (2011). Latent inhibition-related dopaminergic responses in the nucleus accumbens are disrupted following neonatal transient inactivation of the ventral subiculum. Neuropsychopharmacology 36, 1421–1432 10.1038/npp.2011.26 21430650 PMC3096811

[B121] Middleton F. A. Strick P. L. (1996). The temporal lobe is a target of output from the basal ganglia. Proc. Natl. Acad. Sci. U.S.A. 93, 8683–8687 10.1073/pnas.93.16.8683 8710931 PMC38733

[B122] Miller E. K. Cohen J. D. (2001). An integrative theory of prefrontal cortex function. Annu. Rev. Neurosci. 24, 167–202 10.1146/annurev.neuro.24.1.167 11283309

[B123] Mink J. W. (1996). The basal ganglia: focused selection and inhibition of competing motor programs. Prog. Neurobiol. 50, 381–425 10.1016/S0301-0082(96)00042-1 9004351

[B124] Mirolli M. Santucci V. G. Baldassarre G. (2013). Phasic dopamine as a prediction error of intrinsic and extrinsic reinforcement driving both action acquisition and reward maximization: a simulated robotic study. Neural Netw. 39, 40–51 10.1016/j.neunet.2012.12.012 23353115

[B125] Mirolli M. Mannella F. Baldassarre G. (2010). The roles of the amygdala in the affective regulation of body, brain, and behavior. Connect. Sci. 22, 215–245 10.1080/09540091003682553

[B126] Mogenson G. J. Jones D. L. Yim C. Y. (1980). From motivation to action: functional interface between the limbic system and the motor system. Prog. Neurobiol. 14, 69–97 10.1016/0301-0082(80)90018-0 6999537

[B127] Montague P. R. Hyman S. E. Cohen J. D. (2004). Computational roles for dopamine in behavioral control. Nature 431, 760–767 10.1038/nature03015 15483596

[B128] Morris G. Nevet A. Arkadir D. Vaadia E. Bergman H. (2006). Midbrain dopamine neurons encode decisions for future action. Nat. Neurosci. 9, 1057–1063 10.1038/nn1743 16862149

[B129] Mulder A. B. Tabuchi E. Wiener S. I. (2004). Neurons in hippocampal afferent zones of rat striatum parse routes into multi-pace segments during maze navigation. Eur. J. Neurosci. 19, 1923–1932 10.1111/j.1460-9568.2004.03301.x 15078566

[B130] Mushiake H. Saito N. Sakamoto K. Itoyama Y. Tanji J. (2006). Activity in the lateral prefrontal cortex reflects multiple steps of future events in action plans. Neuron 50, 631–641 10.1016/j.neuron.2006.03.045 16701212

[B131] Nachev P. Kennard C. Husain M. (2008). Functional role of the supplementary and pre-supplementary motor areas. Nat. Rev. Neurosci. 9, 856–869 10.1038/nrn2478 18843271

[B132] Niv Y. Daw N. D. Joel D. Dayan P. (2007). Tonic dopamine: opportunity costs and the control of response vigor. Psychopharmacology 191, 507–520 10.1007/s00213-006-0502-4 17031711

[B133] O'Donnell P. Grace A. A. (1995). Synaptic interactions among excitatory afferents to nucleus accumbens neurons: hippocampal gating of prefrontal cortical input. J. Neurosci. 15(5 Pt 1), 3622–3639 7751934 10.1523/JNEUROSCI.15-05-03622.1995 PMC6578219

[B134] O'Keefe J. Burgess N. (1996). Geometric determinants of the place fields of hippocampal neurons. Nature 381, 425–428 10.1038/381425a0 8632799

[B135] O'Keefe J. Nadel L. (1978). The Hippocampus as a Cognitive Map. Oxford: Clarendon Press; New York, NY: Oxford University Press

[B136] O'Reilly R. C. Frank M. J. (2006). Making working memory work: a computational model of learning in the prefrontal cortex and basal ganglia. Neural Comput. 18, 283–328 10.1162/089976606775093909 16378516

[B137] Ostlund S. B. Balleine B. W. (2007a). The contribution of orbitofrontal cortex to action selection. Ann. N.Y. Acad. Sci. 1121, 174–192 10.1196/annals.1401.033 17872392

[B138] Ostlund S. B. Balleine B. W. (2007b). Orbitofrontal cortex mediates outcome encoding in pavlovian but not instrumental conditioning. J. Neurosci. 27, 4819–4825 10.1523/JNEUROSCI.5443-06.2007 17475789 PMC6672090

[B139] Ostlund S. B. Balleine B. W. (2008). Differential involvement of the basolateral amygdala and mediodorsal thalamus in instrumental action selection. J. Neurosci. 28, 4398–4405 10.1523/JNEUROSCI.5472-07.2008 18434518 PMC2652225

[B140a] Otmakova N. Duzel E. Deutch A. Y. Lisman J. E. (2013). The hippocampal-VTA loop: the role of novelty and motivation in controlling the entry of information into long-term memory, in Intrinsically Motivated Learning in Natural and Artificial Systems, eds Baldassarre G. Mirolli M. (Berlin: Springer-Verlag), 235–254 10.1016/j.neuron.2005.05.002

[B141] Packard M. G. Knowlton B. J. (2002). Learning and memory functions of the basal ganglia. Annu. Rev. Neurosci. 25, 563–593 10.1146/annurev.neuro.25.112701.142937 12052921

[B142] Pape H.-C. Paré D. (2010). Plastic synaptic networks of the amygdala for the acquisition, expression, and extinction of conditioned fear. Physiol. Rev. 90, 419–463 10.1152/physrev.00037.2009 20393190 PMC2856122

[B143] Parkinson J. A. Robbins T. W. Everitt B. J. (2000a). Dissociable roles of the central and basolateral amygdala in appetitive emotional learning. Eur. J. Neurosci. 12, 405–413 10.1046/j.1460-9568.2000.00960.x 10651899

[B144] Parkinson J. A. Willoughby P. J. Robbins T. W. Everitt B. J. (2000b). Disconnection of the anterior cingulate cortex and nucleus accumbens core impairs Pavlovian approach behavior: further evidence for limbic cortical-ventral striatopallidal systems. Behav. Neurosci. 114, 42–63 10.1037/0735-7044.114.1.42 10718261

[B145] Pavlides C. Miyashita E. Asanuma H. (1993). Projection from the sensory to the motor cortex is important in learning motor skills in the monkey. J. Neurophysiol. 70, 733–741 8410169 10.1152/jn.1993.70.2.733

[B146] Peciña S. Schulkin J. Berridge K. C. (2006a). Nucleus accumbens corticotropin-releasing factor increases cue-triggered motivation for sucrose reward: paradoxical positive incentive effects in stress? BMC Biol. 4:8 10.1186/1741-7007-4-8 16613600 PMC1459217

[B147] Peciña S. Smith K. S. Berridge K. C. (2006b). Hedonic hot spots in the brain. Neuroscientist 12, 500–511 10.1177/1073858406293154 17079516

[B148] Pennartz C. M. A. Ito R. Verschure P. F. M. J. Battaglia F. P. Robbins T. W. (2011). The hippocampal-striatal axis in learning, prediction and goal-directed behavior. Trends Neurosci. 34, 548–559 10.1016/j.tins.2011.08.001 21889806

[B149] Penner M. Mizumori S. (2011). Neural systems analysis of decision making during goal-directed navigation. Prog. Neurobiol. 96, 96–135 10.1016/j.pneurobio.2011.08.010 21964237

[B150] Peters J. LaLumiere R. T. Kalivas P. W. (2008). Infralimbic prefrontal cortex is responsible for inhibiting cocaine seeking in extinguished rats. J. Neurosci. 28, 6046–6053 10.1523/JNEUROSCI.1045-08.2008 18524910 PMC2585361

[B151] Peterschmitt Y. Hoeltzel A. Louilot A. (2005). Striatal dopaminergic responses observed in latent inhibition are dependent on the hippocampal ventral subicular region. Eur. J. Neurosci. 22, 2059–2068 10.1111/j.1460-9568.2005.04366.x 16262643

[B152] Pezzulo G. Rigoli F. Chersi F. (2013). The mixed instrumental controller: using value of information to combine habitual choice and mental simulation. Front. Psychol. 4:92 10.3389/fpsyg.2013.00092 23459512 PMC3586710

[B153] Preuss T. M. (1995). Do rats have prefrontal cortex? the rose-woolsey-akert program reconsidered. J. Cogn. Neurosci. 7, 1–24 10.1162/jocn.1995.7.1.1 23961750

[B154] Quintero E. Díaz E. Vargas J. P. Casa de la G. López J. C. (2011). Ventral subiculum involvement in latent inhibition context specificity. Physiol. Behav. 102, 414–420 10.1016/j.physbeh.2010.12.002 21147138

[B155] Quirk G. J. Likhtik E. Pelletier J. G. Paré D. (2003). Stimulation of medial prefrontal cortex decreases the responsiveness of central amygdala output neurons. J. Neurosci. 23, 8800–8807 14507980 10.1523/JNEUROSCI.23-25-08800.2003 PMC6740415

[B156] Quirk G. J. Mueller D. (2008). Neural mechanisms of extinction learning and retrieval. Neuropsychopharmacology 33, 56–72 10.1038/sj.npp.1301555 17882236 PMC2668714

[B157] Quirk G. J. Russo G. K. Barron J. L. Lebron K. (2000). The role of ventromedial prefrontal cortex in the recovery of extinguished fear. J. Neurosci. 20, 6225–6231 10934272 10.1523/JNEUROSCI.20-16-06225.2000 PMC6772571

[B158] Ragozzino M. E. (2007). The contribution of the medial prefrontal cortex, orbitofrontal cortex, and dorsomedial striatum to behavioral flexibility. Ann. N.Y. Acad. Sci. 1121, 355–375 10.1196/annals.1401.013 17698989

[B159] Ranganath C. Rainer G. (2003). Neural mechanisms for detecting and remembering novel events. Nat. Rev. Neurosci. 4, 193–202 10.1038/nrn1052 12612632

[B160] Rebec G. V. Christensen J. R. Guerra C. Bardo M. T. (1997). Regional and temporal differences in real-time dopamine efflux in the nucleus accumbens during free-choice novelty. Brain Res. 776, 61–67 10.1016/S0006-8993(97)01004-4 9439796

[B161] Redgrave P. Gurney K. (2006). The short-latency dopamine signal: a role in discovering novel actions? Nat. Rev. Neurosci. 7, 967–975 10.1038/nrn2022 17115078

[B162] Redgrave P. Prescott T. J. Gurney K. (1999). The basal ganglia: a vertebrate solution to the selection problem? Neuroscience 89, 1009–1024 10.1016/S0306-4522(98)00319-4 10362291

[B163a] Redgrave P. Rodriguez M. Smith Y. Rodriguez-Oroz M. C. Lehericy S. Bergman H. (2010). Goal-directed and habitual control in the basal ganglia: implications for parkinson's disease. Nat. Rev. Neurosci. 11, 760–772 10.1038/nrn2915 20944662 PMC3124757

[B164] Rhodes S. E. V. Killcross S. (2004). Lesions of rat infralimbic cortex enhance recovery and reinstatement of an appetitive Pavlovian response. Learn. Mem. 11, 611–616 10.1101/lm.79704 15466316 PMC523080

[B165] Richmond J. Colombo M. (2002). Hippocampal lesions, contextual retrieval, and autoshaping in pigeons. Brain Res. 928, 60–68 10.1016/S0006-8993(01)03355-8 11844472

[B166] Rizzolatti G. Craighero L. (2004). The mirror-neuron system. Annu. Rev. Neurosci. 27, 169–192 10.1146/annurev.neuro.27.070203.144230 15217330

[B167] Roberts A. C. (2006). Primate orbitofrontal cortex and adaptive behavior. Trends Cogn. Sci. 10, 83–90 10.1016/j.tics.2005.12.002 16380289

[B168] Robinson D. L. Venton J. B. Heien M. L. A. V. Wightman M. R. (2003). Detecting subsecond dopamine release with fast-scan cyclic voltammetry *in vivo*. Clin. Chem. 49, 1763–1773 10.1373/49.10.1763 14500617

[B169] Robinson D. L. Wightman M. R. (2004). Nomifensine amplifies subsecond dopamine signals in the ventral striatum of freely-moving rats. J. Neurochem. 90, 894–903 10.1111/j.1471-4159.2004.02559.x 15287895

[B170] Robinson D. L. Zitzman D. L. Williams S. K. (2011). Mesolimbic dopamine transients in motivated behaviors: focus on maternal behavior. Front. Psychiatry. 2:23 10.3389/fpsyt.2011.00023 21629844 PMC3098725

[B171] Roitman M. F. Stuber G. D. Phillips P. E. M. Wightman M. R. Carelli R. M. (2004). Dopamine operates as a subsecond modulator of food seeking. J. Neurosci. 24, 1265–1271 10.1523/JNEUROSCI.3823-03.2004 14960596 PMC6730321

[B172] Rolls E. T. (2000a). Hippocampo-cortical and cortico-cortical backprojections. Hippocampus 10, 380–388 10.1002/1098-1063(2000)10:4<380::AID-HIPO4>3.0.CO;2-0 10985277

[B173] Rolls E. T. (2000b). The orbitofrontal cortex and reward. Cereb. Cortex 10, 284–294 10.1093/cercor/10.3.284 10731223

[B174] Rolls E. T. (2004a). Convergence of sensory systems in the orbitofrontal cortex in primates and brain design for emotion. Anat. Rec. A Discov. Mol. Cell. Evol. Biol. 281, 1212–1225 10.1002/ar.a.20126 15470678

[B175] Rolls E. T. (2004b). The functions of the orbitofrontal cortex. Brain Cogn. 55, 11–29 10.1016/S0278-2626(03)00277-X 15134840

[B176] Rolls E. T. Treves A. (1998). Neural Networks and Brain Function. Oxford: Oxford Unversity Press

[B177] Romanelli P. Esposito V. Schaal D. W. Heit G. (2005). Somatotopy in the basal ganglia: experimental and clinical evidence for segregated sensorimotor channels. Brain Res. Brain Res. Rev. 48, 112–128 10.1016/j.brainresrev.2004.09.008 15708631

[B178] Room P. Russchen F. T. Groenewegen H. J. Lohman A. H. (1985). Efferent connections of the prelimbic (area 32) and the infralimbic (area 25) cortices: an anterograde tracing study in the cat. J. Comp. Neurol. 242, 40–55 10.1002/cne.902420104 4078047

[B179] Runyan J. D. Moore A. N. Dash P. K. (2004). A role for prefrontal cortex in memory storage for trace fear conditioning. J. Neurosci. 24, 1288–1295 10.1523/JNEUROSCI.4880-03.2004 14960599 PMC6730343

[B180] Saito N. Mushiake H. Sakamoto K. Itoyama Y. Tanji J. (2005). Representation of immediate and final behavioral goals in the monkey prefrontal cortex during an instructed delay period. Cereb. Cortex 15, 1535–1546 10.1093/cercor/bhi032 15703260

[B181] Salamone J. D. Correa M. Mingote S. Weber S. M. (2003). Nucleus accumbens dopamine and the regulation of effort in food-seeking behavior: implications for studies of natural motivation, psychiatry, and drug abuse. J. Pharmacol. Exp. Ther. 305, 1–8 10.1124/jpet.102.035063 12649346

[B182] Schoenbaum G. Chiba A. A. Gallagher M. (1998). Orbitofrontal cortex and basolateral amygdala encode expected outcomes during learning. Nat. Neurosci. 1, 155–159 10.1038/407 10195132

[B183] Schoenbaum G. Setlow B. Nugent S. L. Saddoris M. P. Gallagher M. (2003). Lesions of orbitofrontal cortex and basolateral amygdala complex disrupt acquisition of odor-guided discriminations and reversals. Learn. Mem. 10, 129–140 10.1101/lm.55203 12663751 PMC196660

[B184] Schultz W. (2002). Getting formal with dopamine and reward. Neuron 36, 241–263 10.1016/S0896-6273(02)00967-4 12383780

[B185] Schultz W. Dayan P. Montague P. R. (1997). A neural substrate of prediction and reward. Science 275, 1593–1599 10.1126/science.275.5306.1593 9054347

[B186a] Sesack S. R. Deutch A. Y. Roth R. H. Bunney B. S. (1989). Topographical organization of the efferent projections of the medial prefrontal cortex in the rat: an anterograde tract-tracing study with phaseolus vulgaris leucoagglutinin. J. Comp. Neurol. 290, 213–242 10.1002/cne.902900205 2592611

[B187] Shadlen M. N. Newsome W. T. (2001). Neural basis of a perceptual decision in the parietal cortex (area LIP) of the rhesus monkey. J. Neurophysiol. 86, 1916–1936 11600651 10.1152/jn.2001.86.4.1916

[B189] Smith D. M. Mizumori S. J. Y. (2006). Hippocampal place cells, context, and episodic memory. Hippocampus 16, 716–729 10.1002/hipo.20208 16897724

[B190] Solway A. Botvinick M. M. (2012). Goal-directed decision making as probabilistic inference: a computational framework and potential neural correlates. Psychol. Rev. 119, 120–154 10.1037/a0026435 22229491 PMC3767755

[B191] Sotres-Bayon F. Quirk G. J. (2010). Prefrontal control of fear: more than just extinction. Curr. Opin. Neurobiol. 20, 231–235 10.1016/j.conb.2010.02.005 20303254 PMC2878722

[B192] Sutton R. S. Barto A. G. (1987). A temporal-difference model of classical conditioning, in In Proceedings of the Ninth Annual Conference of the Cognitive Science Society (Seattle, WA).

[B193] Sutton R. S. Barto A. G. (1998). Reinforcement Learning: An Introduction. Cambridge MA: The MIT Press

[B194a] Tokimura H. Di Lazzaro V. Tokimura Y. Oliviero A. Profice P. Insola A. (2000). Short latency inhibition of human hand motor cortex by somatosensory input from the hand. J. Physiol. 523(Pt 2), 503–513 10.1111/j.1469-7793.2000.t01-1-00503.x 10699092 PMC2269813

[B195] Toni I. Ramnani N. Josephs O. Ashburner J. Passingham R. E. (2001). Learning arbitrary visuomotor associations: temporal dynamic of brain activity. Neuroimage 14, 1048–1057 10.1006/nimg.2001.0894 11697936

[B196] VanElzakker M. Fevurly R. D. Breindel T. Spencer R. L. (2008). Environmental novelty is associated with a selective increase in fos expression in the output elements of the hippocampal formation and the perirhinal cortex. Learn. Mem. 15, 899–908 10.1101/lm.1196508 19050162 PMC2632843

[B197] Vertes R. P. (2004). Differential projections of the infralimbic and prelimbic cortex in the rat. Synapse 51, 32–58 10.1002/syn.10279 14579424

[B198] Voorn P. Vanderschuren L. J. M. J. Groenewegen H. J. Robbins T. W. Pennartz C. M. A. (2004). Putting a spin on the dorsal-ventral divide of the striatum. Trends Neurosci. 27, 468–474 10.1016/j.tins.2004.06.006 15271494

[B199] Watkins C. J. Dayan P. (1992). Q-learning. Mach. learn. 8, 279–292 10.1023/A:1022676722315

[B200] Wise S. P. (2008). Forward frontal fields: phylogeny and fundamental function. Trends Neurosci. 31, 599–608 10.1007/BF00992698 18835649 PMC2587508

[B201] Wittmann B. C. Bunzeck N. Dolan R. J. Düzel E. (2007). Anticipation of novelty recruits reward system and hippocampus while promoting recollection. Neuroimage 38, 194–202 10.1016/j.neuroimage.2007.06.038 17764976 PMC2706325

[B202] Wyvell C. L. Berridge K. C. (2000). Intra-accumbens amphetamine increases the conditioned incentive salience of sucrose reward: enhancement of reward “wanting” without enhanced “liking” or response reinforcement. J. Neurosci. 20, 8122–8130 11050134 10.1523/JNEUROSCI.20-21-08122.2000 PMC6772712

[B203] Yin H. H. Knowlton B. J. (2006). The role of the basal ganglia in habit formation. Nat. Rev. Neurosci. 7, 464–476 10.1038/nrn1919 16715055

[B204] Yin H. H. Knowlton B. J. Balleine B. W. (2004). Lesions of dorsolateral striatum preserve outcome expectancy but disrupt habit formation in instrumental learning. Eur. J. Neurosci. 19, 181–189 10.1111/j.1460-9568.2004.03095.x 14750976

[B205] Yin H. H. Ostlund S. B. Balleine B. W. (2008). Reward-guided learning beyond dopamine in the nucleus accumbens: the integrative functions of cortico-basal ganglia networks. Eur. J. Neurosci. 28, 1437–1448 10.1111/j.1460-9568.2008.06422.x 18793321 PMC2756656

[B206] Yin H. H. Ostlund S. B. Knowlton B. J. Balleine B. W. (2005). The role of the dorsomedial striatum in instrumental conditioning. Eur. J. Neurosci. 22, 513–523 10.1111/j.1460-9568.2005.04218.x 16045504

[B207] Zahm D. S. (2000). An integrative neuroanatomical perspective on some subcortical substrates of adaptive responding with emphasis on the nucleus accumbens. Neurosci. Biobehav. Rev. 24, 85–105 10.1016/S0149-7634(99)00065-2 10654664

